# Tuning the Morphology and Structure of Supraparticles Composed of Ellipsoids

**DOI:** 10.1021/acs.langmuir.5c00409

**Published:** 2025-03-10

**Authors:** Melis Yetkin, Yashraj Manish Wani, Arash Nikoubashman, Hans-Jürgen Butt, Michael Kappl

**Affiliations:** † Department of Physics at Interfaces, Max-Planck Institute for Polymer Research, 55128 Mainz, Germany; ‡ Institute of Physics, Johannes Gutenberg University of Mainz, Staudingerweg 7, 55128 Mainz, Germany; § Leibniz-Institut für Polymerforschung Dresden e.V., Hohe Straße 6, 01069 Dresden, Germany; ∥ Institut für Theoretische Physik, Technische Universität Dresden, 01069 Dresden, Germany

## Abstract

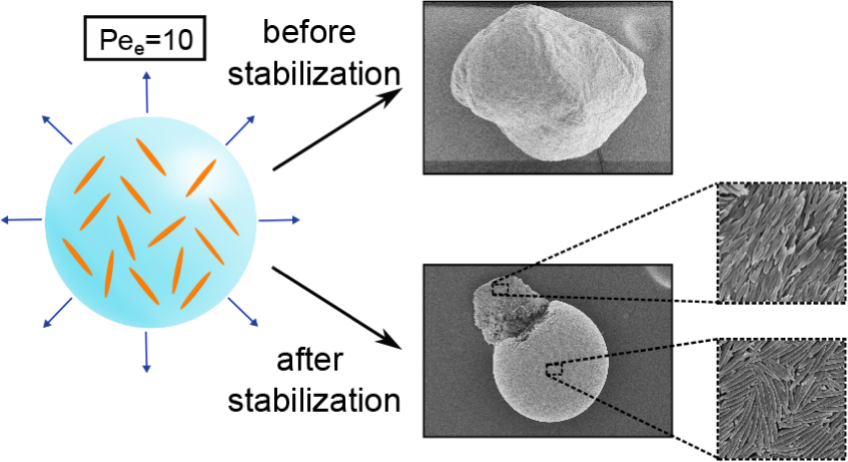

Evaporating droplets of colloidal dispersions on superamphiphobic surfaces is a versatile way to fabricate supraparticles with complex shape and internal morphology. Here, we investigated experimentally the formation of supraparticles consisting of polystyrene (PS) ellipsoids, with aspect ratios ranging from 4 to 11, and of sphere–ellipsoid mixtures. We demonstrated that the final supraparticle morphology can be tuned from buckled to spherical through the addition of the surfactant sodium dodecyl sulfate (SDS), which stabilizes the colloids within the drying droplet. At low evaporation speeds, we observed the ordering of the ellipsoidal colloids into small nematic bundles inside the supraparticle and into smectic domains on the supraparticle surface, which is consistent with results from previous computer simulations. The orientational ordering of ellipsoidal colloids was disrupted in the binary supraparticles consisting of sphere–ellipsoid mixtures, even at low sphere concentrations.

## Introduction

Drying droplets of colloidal dispersions can result in the formation of assembled micron to millimeter-sized particles, which are termed supraparticles.^1,2^ The properties and functionalities of supraparticles can be engineered through the size, shape, chemistry, and arrangement of their colloidal building blocks.^1,3^ Traditionally, spherical colloids have been used to produce supraparticles of various structures and compositions.^4−7^ Using nonspherical (anisotropic) colloids as building blocks adds new opportunities in tuning the structure and function of supraparticles. For example, supraparticles as optoelectronic materials^8^ and scattering enhancers^9^ were proposed by controlling the ordering of their lanthanide fluoride nanoplatelet and silica nanorod building blocks, respectively. Supraparticles with a more open, porous structure were created using cubic hematite nanocrystals for gas sensing and photocatalysis^10^ and using silica-coated gold nanorods for enhanced Raman scattering.^11^

However, controlling and predicting the assembly of anisotropic colloids into supraparticles is more complex because of their additional orientational degrees of freedom and possible interaction anisotropy.^12−14^ For hard particles, the phase behavior is dictated by excluded volume effects that lead to depletion interactions,^15−17^ whose strength and range depend on the size, aspect ratio, and the concentration of the particles.^18,19^ Phase separation of sphere-rod mixtures has already been observed both experimentally and theoretically in bulk systems at equilibrium.^15,18,20−22^ Confining anisotropic colloids may influence their orientational ordering and phase behavior, which in turn could affect the emerging functional material properties.^23−28^ For example, spherical colloids were shown to destabilize the orientational ordering of rods in simulation studies of sphere-rod mixtures confined between two hard walls.^14^ This phase separation behavior can be used in spherical confinement to make multicomponent supraparticles, e.g., Janus supraparticles.^29^

Supraparticles can be formed by drying colloidal dispersion droplets on superamphiphobic surfaces. These droplets assume a nearly spherical droplet morphology with high contact angles (>150°) and extremely small interfacial area between liquid and solid (≪*V*^2/3^, where *V* is the droplet volume).^30−32^ Therefore, the evaporation of droplets on superamphiphobic surfaces is almost radially symmetric^33^ as in Leidenfrost levitation^34,35^ or spray-drying.^36−38^ Using superamphiphobic surfaces has the advantage that evaporation occurs at much longer time scales so that structure formation can be studied in detail under well-controlled conditions.^33^ Furthermore, this technique does not require the use and disposal of additional processing liquids,^39^ as is the case for microfluidics^40^ or emulsion evaporation.

The evaporation-induced self-assembly of colloids can be characterized by a dimensionless quantity known as the Péclet number, *Pe*. It is defined as the ratio of the typical diffusion time and the evaporation time at the beginning of drying, *Pe* = τ_d_/τ_ev_ = *v*_ev_*R*_0_/*D*_0_, where *R*_0_ is the initial droplet radius, *D*_0_ is the diffusion coefficient of the colloidal particles at infinite dilution, and *v*_ev_ is the initial speed of the receding droplet interface. We define *Pe* ≫ 1 as the evaporation-limited regime and *Pe* ≤ 1 as the diffusion-limited regime. For *Pe* ≫ 1, evaporation is fast compared to the diffusion of the confined colloids, so that steep concentration gradients might develop within the drying droplet. The resulting elastic shell formed during drying then eventually buckles due to capillary stress, leading to supraparticles with a crumpled irregular shape.^35,41^ When *Pe* ≤ 1, the droplet typically shrinks isotropically and retains a homogeneous colloid distribution throughout drying, thus facilitating the formation of spherical supraparticles.

The final supraparticle morphology is not only determined by the kinetics of drying, but also by the interplay among several parameters such as interparticle interactions, the initial colloid volume fraction, the colloid size, and the viscosity of the colloidal dispersion.^41−43^ Tsapis et al. promoted buckling for levitated polystyrene (PS) colloidal droplets by decreasing the net repulsion between the colloids by adding salt.^35^ Conversely, Sen et al. suppressed buckling of spray-dried silica suspensions through the addition of the surfactant sodium dodecyl sulfate (SDS).^44,45^ Prior research indicated the role of primary particle shape in controlling buckling. Al Harraq and Bharti observed suppression of buckling for supraparticles containing high aspect ratio (>9) rods, contrasted by the buckling for supraparticles containing rods of lower aspect ratio (<9).^46^ Prevention of buckling was attributed to more permeable shells formed by more elongated rods. In another study, Mondal et al. formed buckled, doughnut-shaped supraparticles with ellipsoids of aspect ratio 4. In contrast, spherical supraparticles formed when using ellipsoids of aspect ratio 2.^47^

The final desired supraparticle morphology, e.g., spherical or buckled, depends on the type of application. For instance, buckled supraparticles might be more suitable for catalysis and absorption applications due to larger surface-to-volume ratios compared to spheres, whereas spherical supraparticles might be used as drug encapsulators.^42^ Therefore, fine-tuning the above-mentioned variables is important to control the final morphology of supraparticles.

In a previous study, we investigated the formation of supraparticles from ellipsoidal colloids and from sphere–ellipsoid mixtures.^48^ Diffusion-limited drying simulations (*Pe* ≤ 1) showed a strong increase in the orientational ordering of rods on the supraparticle surface and in the local nematic ordering within the interior. Furthermore, the relative area fraction of ellipsoidal colloids (σ_e_) on the binary supraparticle surfaces decreased from 82% ≤ σ_e_ ≤ 96% to about 50% with increasing evaporation rate for droplets containing an equal volume ratio of both colloid types.^48^ In this study, we advance the design rules for the production of supraparticles composed of ellipsoidal colloids, taking into account important aspects such as colloidal stability, the evaporation rate, and the volume ratio of the components in the case of binary mixtures. Our spherical and ellipsoidal PS colloids have similar interaction potentials and surface chemistry, which enabled us to better focus on the physical processes of supraparticle formation. We found that (i) buckling occurred irrespective of the primary particle shape when the colloidal dispersions were not stabilized with surfactant; (ii) at low Péclet numbers, the ordering of ellipsoidal colloids on the surface and interior of the supraparticles improved compared to that at high Péclet numbers; (iii) for the binary mixtures, spherical colloids suppressed the surface and interior ordering of ellipsoidal colloids even at low sphere concentrations and at low Péclet numbers.

## Materials and Methods

### Materials

Methyl trichlorosilane (TCMS, 99%, Sigma-Aldrich), hexadecane (Reagent Plus, 99%, Sigma-Aldrich), and 1*H*,1*H*,2*H*,2*H*-perfluorodecyltrichlorosilane (PFDTS, 96%, Alfa Aesar) were used in the preparation of superamphiphobic surfaces. *N*-Hexane (≥95%), toluene (≥99.8%), acetone (≥99.8%), and isopropyl alcohol (IPA, ≥ 99.8%) were purchased from Fisher Chemical. Ethanol (≥99.8%) was provided by Honeywell Research Chemicals. Ultrapure water with a resistivity of 18.2 MΩ·cm was obtained by using a Sartorius Arium 611 VF water purification system. Glass slides, 25 × 75 mm^2^ in size, were provided by Menzel-Gläser, Germany. PS colloids (negatively charged ≈ −59 mV zeta potential, with −COOH groups on the surface) with a diameter of either 408 or 450 nm were synthesized by the copolymerization of styrene and acrylic acid using surfactant-free emulsion polymerization.^49^ The synthesized colloids were purified by several centrifugation cycles and were finally redispersed in ultrapure water. Poly(vinyl alcohol) (PVA, molecular weight 115 kg/mol, degree of hydrolysis 86.5%–89%) was purchased from VWR Chemicals. Anionic surfactant SDS (molecular weight of 288.4 g/mol, MP Biomedicals) was used to stabilize the colloidal dispersions. The aqueous dispersion of fluorescently labeled spherical PS colloids (carboxylate-modified, fluorescent red, diameter 500 nm) was provided by Sigma-Aldrich.

### Preparation of Superamphiphobic Surfaces

The silicone nanofilament-based superamphiphobic surfaces were prepared by the grow-from method, as described previously.^48,50,51^ In short, cleaned glass slides were first activated by oxygen plasma at 30 W for 2 min (Diener Electronic Femto). To initiate the growth of silicone nanofilaments, the glass slides were then immersed in a sealed reaction chamber, which contained a mixture of toluene (360 mL, 166 ppm water) and TCMS (250 μL) for 14 h. The water content of toluene was measured with a Karl Fischer coulometer (Mettler Toledo C20 Compact KF coulometer). After the reaction, the glass slides were again activated with oxygen plasma at 120 W for 2 min and were further modified with PFDTS (100 μL) in hexane (120 mL) for 30 min to achieve superamphiphobicity. Water and hexadecane droplets acquired spherical shapes on the resultant nanofilament-coated glass substrates. The coating was homogeneous over a large area.

### Preparation of Ellipsoidal Colloids

Ellipsoidal PS colloids were created by a well-established film-stretching method,^52^ in which monodisperse spherical PS colloids were embedded in a PVA matrix film and stretched in an oven above the glass transition temperature of the colloids (for details, see Yetkin et al.^48^). A homogeneous PVA/colloid dispersion was prepared by stirring a 3.5 wt % aqueous PVA solution with 4 g of 10 wt % colloidal PS dispersion at 375 rpm for 5 h. The weight ratio of PS to PVA in the final mixture was 0.08. The PVA/colloid dispersion was then air-dried for 1 day from a flat Teflon mold (15 × 28 cm^2^). The colloid-embedded PVA film was then stretched by applying uniaxial tension with a tensile test apparatus (Zwick/Roell Z005 Universal Testing Machine) in an oven at 140 °C. The films were stretched at draw ratios of 100, 200, and 400% to obtain ellipsoidal colloids with varying aspect ratios (λ) of about 4, 6, and 11, respectively. The size and aspect ratio were calculated by counting 40 colloids in a scanning electron microscopy (SEM) image of each batch (Table 1). The PVA matrix was dissolved in water via several washing steps to recover the ellipsoidal PS colloids from the film. The resultant colloids were finally dispersed in an appropriate amount of distilled water depending on the required colloid concentration.

**Table 1:** Dimensions of the Ellipsoidal Colloids Obtained by Stretching a Film Embedded with Either 408 nm* or 450 nm** PS Spherical Colloids^a^

Draw ratio (%)	*L* (μm)	*d*_e_ (μm)	λ = *L*/*d*_e_
100*	0.92 ± 0.05	0.26 ± 0.01	3.5 ± 0.3
200*	1.30 ± 0.11	0.21 ± 0.02	6.1 ± 0.8
400*	1.95 ± 0.21	0.18 ± 0.02	11 ± 2
200**	1.37 ± 0.10	0.19 ± 0.02	7.2 ± 0.9

a The dimensions (length *L* and diameter *d*_e_) were determined by analyzing 40 colloids each.

### Evaporation of PS Dispersion Droplets on Superamphiphobic Surfaces

The volume fraction of colloidal PS ellipsoids or spheres in the nonstabilized dispersions was kept around 1%. The stabilized dispersions contained 1 mM aqueous SDS solution and had a volume concentration of 0.5% PS ellipsoids. In the case of sphere–ellipsoid mixture dispersions, the total volume concentration varied between 0.6% and 1% with volume ratios of ellipsoids to spheres of *v*_e_:*v*_s_ = 0.4, 1, 2.5, 5, and 10. An Eppendorf Research Plus pipette, which was equipped with epT.I.P.S. LoRetention Reloads tips, was used to dispense 1 μL of these dispersions onto superamphiphobic surfaces. Evaporation experiments were performed inside an in-house-built humidity chamber and were recorded by a camera (Blackfly S Color 5.0 MP USB3 Camera). The relative humidity (RH) during the evaporation of the dispersion droplets was recorded with a humidity sensor inside the chamber. Once the humidity inside the chamber equilibrated, the droplets were dispensed through the holes on top of the chamber, which remained closed throughout the evaporation. The dispersion droplets evaporated at 23 °C, either under ambient conditions (RH = 30%) or inside the chamber at RH = 75% with regulated nitrogen flow. Additional evaporation experiments were performed at RH = 90% by placing several water containers around the superamphiphobic substrate inside the sealed chamber. It should be noted here that the humidity value ranged between 90% and 95% and was no longer precise under these conditions due to the limitations of the humidity sensor (the allowed range is typically between 10% and 90%). The final supraparticles were collected in small vials by slightly tilting the surfaces to make them roll off. The evaporation experiments were repeated at least twice for each system presented.

### Characterization

The structure of the supraparticles was imaged by SEM (Hitachi SU8000 Type 1). The samples were sputter-coated before imaging with a 7 nm layer of Pt by using a Safematic Compact Coating Unit-010 to avoid charging. The zeta potential of the primary PS colloids was measured by a Malvern Zetasizer Nano Z. Using ImageJ software, we performed image analysis with the evaporation videos of the droplets and recorded the change in droplet volume as a function of time. Péclet numbers were calculated by estimating the droplet volume from the evaporation videos using the solid of revolution method, as described in our previous study^48^ (see the Supporting Information for details and Figure S1 for evaporation plots). For some experiments, we used a laser scanning confocal microscope (Leica Microsystems TCS SP8) to study the changes in the inner structure during the drying process. In order to do that, 1 μL dispersion drop was dispensed on a transparent superamphiphobic surface, which was coated on a microscope cover glass (Carl Roth GmbH, thickness: 170 ± 5 μm). The confocal microscope was operated with a dry objective 10× at 4.5 or 2.5 zoom and with an excitation at 514 nm using an Ar laser.

## Results and Discussion

### Supraparticle Formation with Buckling

**Figure 1:**
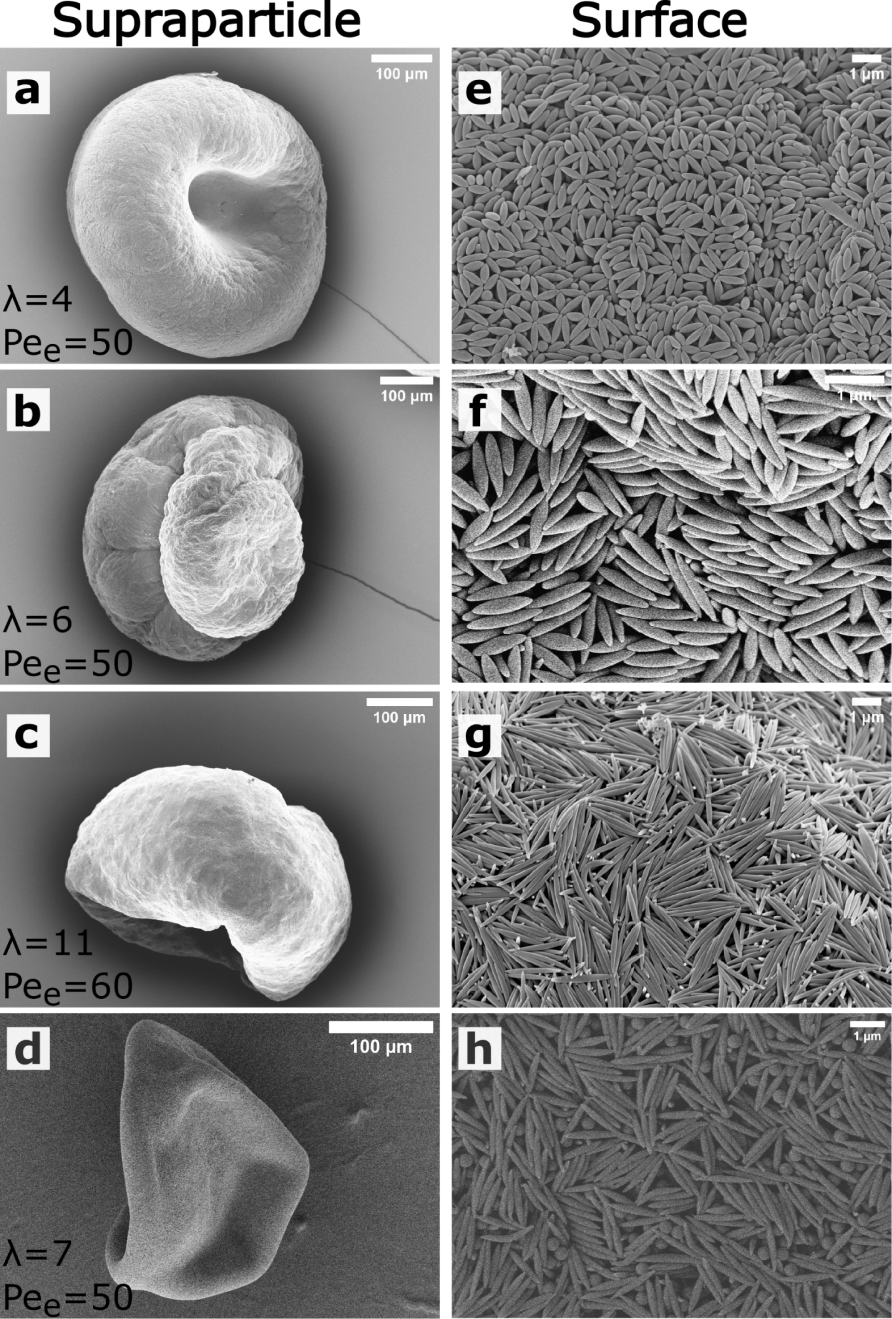
SEM images of supraparticles and their surfaces, comprising ellipsoidal colloids of (a,e) λ = 4, (b,f) λ = 6, (c,g) λ = 11, and (d,h) comprising a sphere–ellipsoid mixture with ellipsoidal colloids of λ = 7 dried at 50 ≤ *Pe*_e_ ≤ 60.

Aqueous dispersion droplets (with initial radii of about *R*_0_ ≈ 620 μm and final supraparticle radii of about *R*_f_ ≈ 100 μm) containing PS ellipsoids of varying aspect ratios (λ ≈ 4, 6, and 11, with lengths 0.92, 1.30, and 1.95 μm, Table 1) were dried on superamphiphobic surfaces at RH = 75%. The diffusion coefficients of ellipsoids were determined as described in the Supporting Information and were 5.96 × 10^–13^ m^2^ s^–1^ ≤ *D*_e_ ≤ 5.30 × 10^–13^ m^2^ s^–1^ with increasing aspect ratio. In the following text, *Pe*_e_ and *Pe*_s_ refer to the Péclet numbers for the ellipsoidal and spherical colloids, respectively. The evaporation experiments were performed inside a humidity chamber, in which the humidity was kept constant by allowing a continuous nitrogen flow (see the Materials and Methods section for details). The complete drying of the droplets under these conditions took almost 90 min, leading to 50 ≤ *Pe*_e_ ≤ 60. The obtained supraparticles had an irregular final shape that differed for each aspect ratio (Figures 1a–c and S2). Each supraparticle featured at least one dimpled region and wrinkled surface texture. The ellipsoidal colloids locally formed laterally aligned bundles on the supraparticle surface (Figure 1e–g). Most of the ellipsoids oriented in-plane for λ = 4 and 6, whereas some ellipsoids aligned out-of-plane for λ = 11. However, we did not notice any significant changes in the local ordering of ellipsoids around the upright ones. The supraparticles were cut with the help of a scalpel to image their internal structure by SEM. The ellipsoids of all aspect ratios were randomly oriented inside the supraparticles (Figure S3).

**Figure 2:**
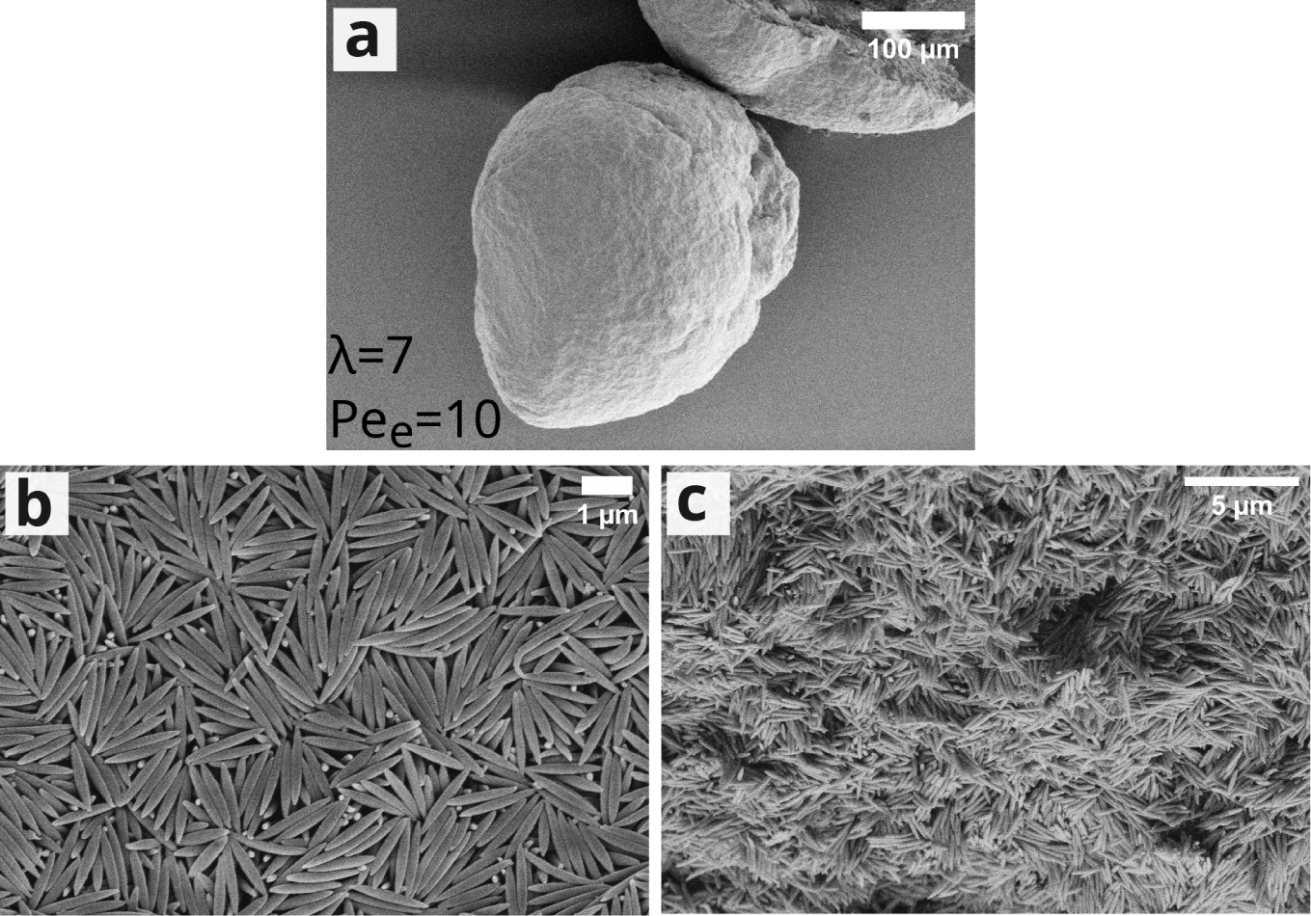
SEM images showing (a) the final supraparticle, (b) supraparticle surface, and (c) supraparticle cross-section, composed of λ = 7 ellipsoids dried at *Pe*_e_ = 10.

To probe the effect of the evaporation rate on the final supraparticle structure, we performed drying experiments at lower and higher relative humidity. When decreasing the humidity, evaporation became faster and took about 30 min (RH = 30%, 170 ≤ *Pe*_e_ ≤ 200). Again, supraparticles with a similarly crumpled final shape (Figure S4) and wrinkled surface were obtained (Figure S5). Likewise, ellipsoidal colloids formed small regions of laterally oriented bundles on the supraparticle surface. For the highest relative humidity, the complete drying of the dispersion droplets of ellipsoidal PS colloids (λ = 4 and 7) took almost four times longer compared to those shown in Figure 1 (315 min, at RH = 90%, leading to *Pe*_e_ = 10). However, the obtained supraparticle morphology was still irregular with a wrinkled surface (Figures 2a, S6, and S7). The ellipsoidal colloids on the supraparticle surface (Figures 2b, S6, and S7) appeared more aligned compared to those at 50 ≤ *Pe*_e_ ≤ 60, while the ellipsoidal colloids inside the supraparticles were still randomly oriented (Figures 2c, S6, and S7).

**Figure 3:**
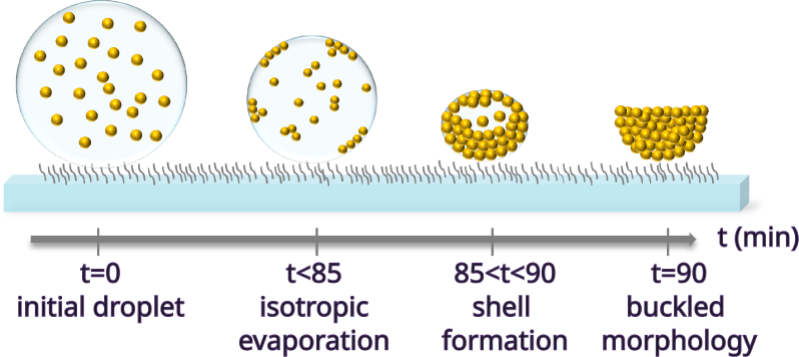
Schematic illustration showing the buckling process of an evaporating droplet on a superamphiphobic surface.

The deviation from spherical supraparticle morphology is a common phenomenon for drying dispersion droplets caused by mechanical instabilities, such as fracture or buckling.^35,53,54^ During the initial period of evaporation, when the colloid concentration is still relatively low, the droplets behave like a pure liquid and shrink isotropically. As more water is evaporated from the droplet, the overall colloid concentration increases. In the diffusion-limited regime (*Pe* ≤ 1), there is sufficient time for the colloids to equalize concentration gradients through diffusion. For large Péclet numbers, however, the interface shrinks faster than the colloids can diffuse, resulting in denser colloid layers close to the droplet–air interface. Toward the end of evaporation, the thickening shell layer prevents the droplet from shrinking isotropically. Instead, the supraparticles develop a flattened region at their bottom and later take on a deflated irregular shape. During this stage, attractive capillary forces will come into play, which may further densify the layer and finally cause buckling of the shell to relieve its stress, as shown schematically in Figure 3.^55−57^ Since ellipsoidal colloids exhibit strong lateral capillary interactions depending on their aspect ratio and contact angle,^58,59^ they may easily drive the deformation of a shell during evaporation.

To observe how the primary colloid shape affects the final supraparticle morphology, we performed similar drying experiments using spherical PS colloids at *Pe*_s_ = 10 (1% initial volume fraction, diameter 408 nm, at RH = 75% with the diffusion coefficient of spheres estimated as *D*_s_ = 2.17 × 10^–12^ m^2^ s^–1^). The supraparticles composed of PS spheres showed a small buckled region from the top but remained otherwise spherical (Figure S8). The surface had a rather smooth texture which consisted of crystalline regions with some line and dot defects. Increasing the initial volume fraction of colloidal particles was previously shown to hinder buckling due to improved mechanical stability.^60,61^ Likewise, we observed only a small buckled region when supraparticles were prepared using droplets of PS spheres with an increased initial volume fraction (8%) (Figure S9). Thus, ellipsoidal colloids enhance shell formation and buckling.

Finally, we fabricated supraparticles by evaporating dispersion droplets of sphere–ellipsoid mixtures (0.6% initial volume fraction, with *v*_e_:*v*_s_ = 1 and at RH = 75%). The obtained supraparticles (consisting of 450 nm spherical colloids and λ = 7 ellipsoidal colloids, with a corresponding length of about 1.37 μm and *D*_e_ = 6.10 × 10^–13^ m^2^ s^–1^, under *Pe*_e_ = 50 and *Pe*_s_ = 10) were ill-shaped and showed buckling from the top (Figure 1d). The surface of the binary supraparticles was mostly covered with randomly oriented ellipsoidal colloids with some spherical colloids in the interstitial spaces (Figure 1h). Hence, the presence of spherical colloids hinders the alignment of ellipsoidal colloids.

**Figure 4:**
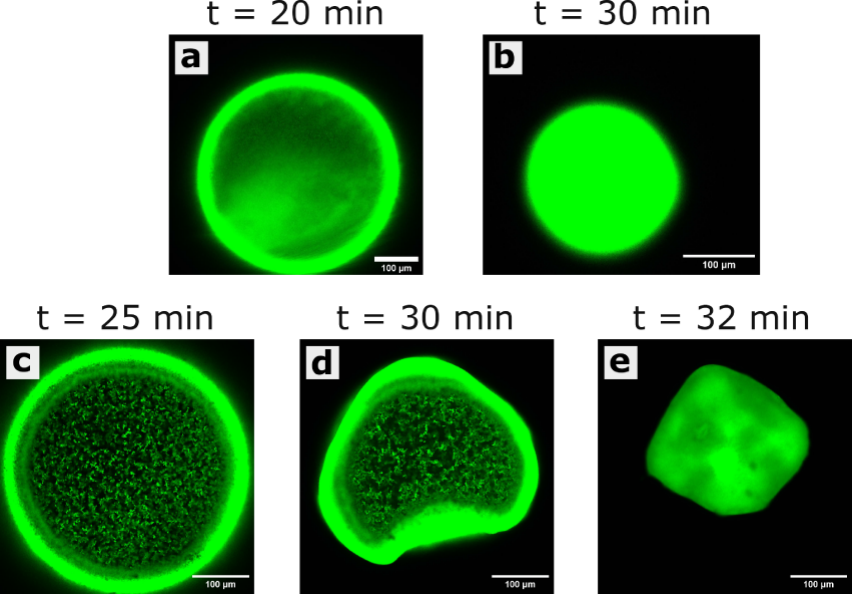
Confocal microscopy snapshots of an evaporating droplet of fluorescently labeled commercial PS spheres after (a) 20 min and (b) the resultant supraparticle after 30 min. Snapshots of an evaporating droplet of a sphere–ellipsoid mixture (with ellipsoids of λ = 4) after (c) 25 min, (d) 30 min, and (e) the resultant supraparticle after 32 min. The mixture droplet contained fluorescently labeled commercial PS spheres and nonfluorescent ellipsoids of an equal volume ratio of the constituents.

Details of the evaporation process were monitored by laser scanning confocal microscopy to better visualize the distribution of colloids within the droplet during buckling. One μL of dispersion droplets containing fluorescently labeled spherical PS colloids (500 nm in diameter, 0.1% initial volume fraction) and mixtures of these labeled spheres with nonlabeled ellipsoidal colloids (λ = 4 ellipsoids, 0.2% initial volume fraction, and *v*_e_:*v*_s_ = 1) were imaged during evaporation at ambient conditions (RH = 30%). The slower evaporation experiments were not monitored since the confocal microscope could not be coupled with a humidity chamber. The initial volume fractions of colloidal particles in the droplets used in the confocal experiments were kept much more dilute to prevent refractive index mismatch. Although the droplets containing only spheres showed a distinct accumulation of colloids at the droplet–air interface during drying (Figure 4a), the final supraparticles obtained after about 30 min were still almost perfectly spherical (Figure 4b). Similarly, a dense layer of colloids at the droplet–air interface also formed for the sphere–ellipsoid mixtures, which was accompanied by a prominent jamming of both fluorescently labeled spheres and nonlabeled ellipsoids in the core (Figure 4c). Then, the spherical shell started deforming (Figure 4d), resulting in a highly irregular supraparticle morphology once the colloids consolidated at the end of drying (Figure 4e).

### Supraparticle Formation with Suppressed Buckling

In previous experiments, Liu et al.^62^ observed that the addition of salt induced buckling in drying dispersion droplets containing spherical PS colloids. This behavior was linked to the screening of the electrostatic repulsion between colloids, which in turn caused the formation of small colloidal aggregates during drying. These aggregates have much smaller mobility compared to that of individual colloids, which facilitates the buildup of concentration gradients within the drying droplet. Further, the hydrophobic nature of PS colloids might also promote the formation of a skin layer at the receding droplet–air interface, which could accelerate buckling. In our experiments, the surface of the PS colloids carried charged −COOH groups, resulting in a zeta potential of ≈−59 mV, which potentially stabilizes them against aggregation due to van der Waals forces.^62−64^ However, the electrostatic double-layer repulsion did not seem to be strong enough to prevent aggregation at high colloid concentrations.

To increase the repulsive interaction between the colloids, we added the anionic surfactant SDS to each dispersion. We hypothesize that the hydrophobic alkyl tail of the SDS molecule adsorbs onto the hydrophobic PS colloids, while the polar sulfate headgroup enhances the electrostatic repulsion between the colloids. Previous colloidal probe atomic force microscopy measurements with hydrophobic particles showed such an increase in electrostatic repulsion upon adding SDS to an aqueous electrolyte.^65^ A similar enhancement in electrostatic repulsion and improvement in colloidal stability has also been confirmed through light scattering, which showed increased Fuchs stability ratios following the addition of ionic surfactants.^66−68^

**Figure 5:**
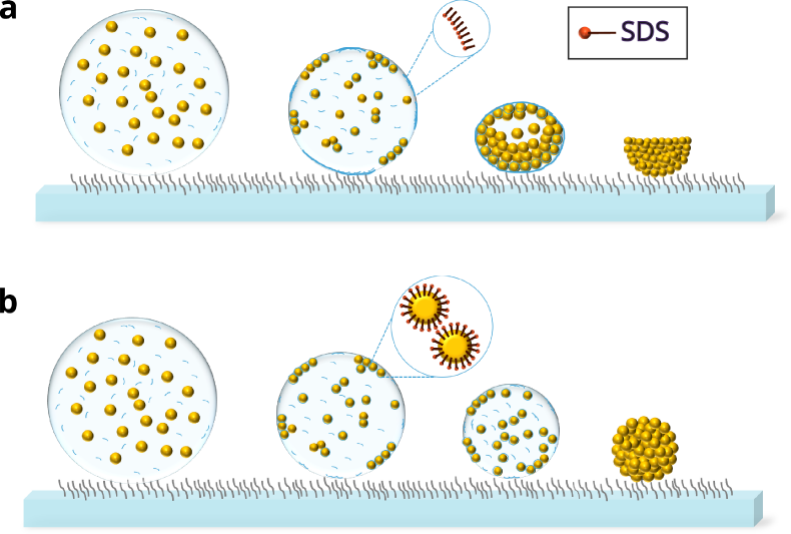
Schematic showing (a) buckling of an evaporating droplet on the superamphiphobic surface in case SDS adsorbs to the droplet–air interface to decrease its surface tension and (b) suppression of buckling in case SDS delays aggregation of colloids and shell formation due to increase in electrostatic repulsion.

Adding SDS to our dispersion droplets can have two competing effects: on the one hand, SDS could adsorb to the droplet surface, with the hydrocarbon chains oriented toward the vapor phase,^69^ and reduce its surface tension. This reduction would promote buckling (Figure 5a), as the formation of a larger surface area becomes less energetically costly due to the reduced surface free energy. On the other hand, SDS may adsorb to the colloid surface and increase its effective charge. As a result, the electrostatic double-layer repulsion becomes stronger, delaying shell formation and thus preventing buckling (Figure 5b). In both scenarios, the SDS concentration increases as water evaporates from the droplet. Once it exceeds the critical micelle concentration (which is about 8 mM for SDS^70^), the nonadsorbed surfactant molecules form micelles, which induce depletion attraction between the colloids and consequently stimulate their aggregation. It is therefore important to add enough ionic surfactant to increase the electrostatic double-layer repulsion between the colloids without exceeding levels that could trigger the undesired side effects described above.

To determine the initial SDS concentration required to stabilize the dispersions, we performed several trial experiments, taking into account the increase in surface area of an ellipsoidal colloid relative to a spherical particle with the same volume. The PS spheres were stabilized with 0.1 mM SDS, so we increased the SDS concentration to 0.15, 0.175, and 0.26 mM for ellipsoids with λ = 4, 6, and 11, respectively. However, these estimated amounts of SDS were insufficient to prevent buckling, particularly for λ ≥ 6. Therefore, we increased the SDS concentration to 1 mM SDS, for which all dispersions remained stable. Throughout the evaporation process, the SDS concentration increased up to 240 mM, which was estimated by considering the initial droplet volume (1 μL), the initial SDS concentration inside the droplet (1 mM), and the final droplet volume (4.2 × 10^–3^ μL).

**Figure 6:**
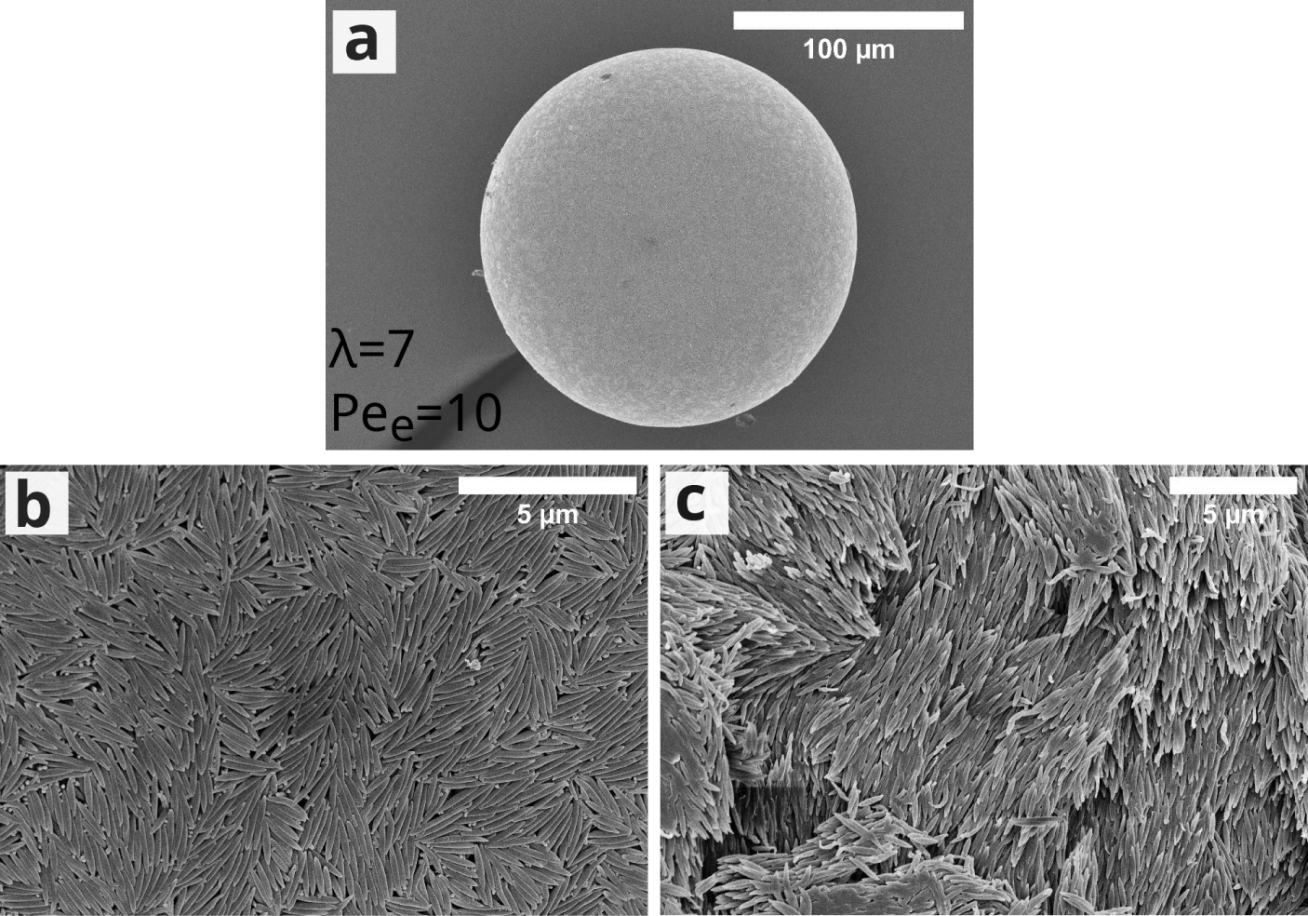
SEM images showing (a) the final supraparticle, (b) supraparticle surface, and (c) supraparticle cross-section, composed of SDS-added dispersion droplets with λ = 7 ellipsoids dried at *Pe*_e_ = 10.

We performed evaporation experiments at *Pe*_e_ = 10 with SDS-added dispersion droplets of PS ellipsoidal colloids (λ = 4 and 7, 0.5% initial volume fraction, RH = 90%). After the addition of SDS, the supraparticles showed an almost spherical final morphology (Figures 6a, S10, and S11), suggesting that the mechanism shown in Figure 5b dominates the supraparticle formation. In addition, the degree of surface (Figures 6b, S10, and S11) and internal ordering (Figures 6c, S10, and S12) of the ellipsoidal colloids increased.

The extent of lateral ordering of the ellipsoidal colloids on the supraparticle surface was quantified by computing the orientational order parameter *S*^†^ (eq 1), as described in our previous study,^48^
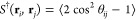
1where θ_*ij*_ ∈ [0, π/2] is the angle between the long axes of two ellipsoids *i* and *j*, whose centers of mass are separated by distance *r* = |**r**_*j*_ – **r**_*i*_|, with **r**_*i*_ and **r**_*j*_ being the positions of ellipsoids *i* and *j*, respectively. A fully parallel alignment of the ellipsoids would lead to *S*^†^ = 1, whereas a random configuration would result in *S*^†^ = 0. The orientational order parameter was calculated only for ellipsoids lying planar on the supraparticle surface. In contrast, the ordering of ellipsoidal colloids inside the supraparticles could only be assessed visually, since the supraparticle cross-sections, prepared by cutting, had a rough and nonplanar interface due to random breakage of the supraparticles.

**Figure 7:**
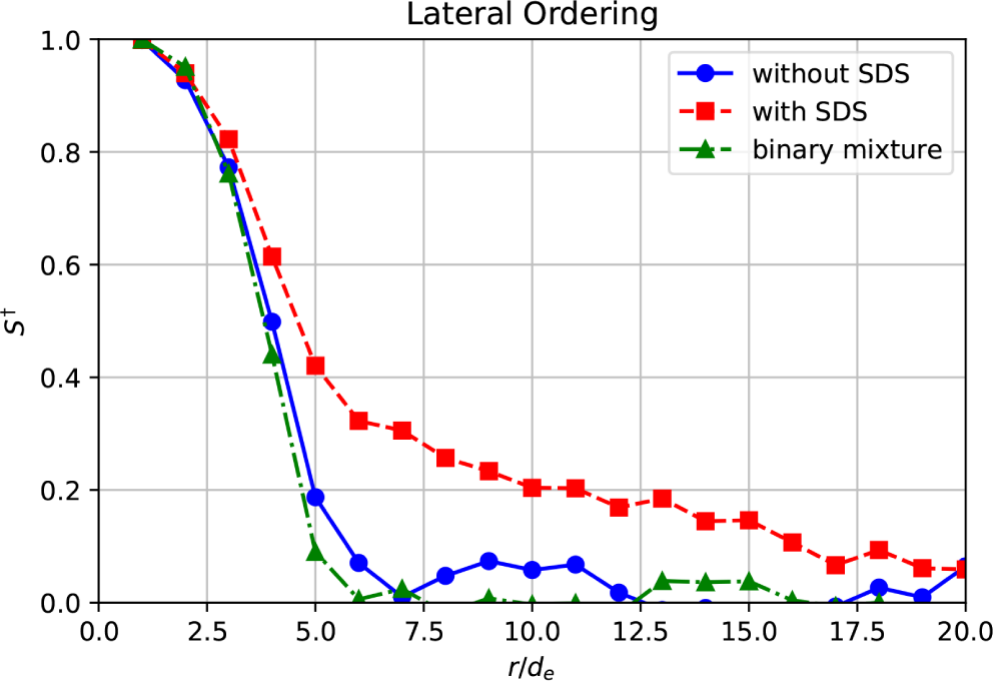
Lateral ordering *S*^†^ between two ellipsoidal colloids (λ = 7) with center-to-center distance *r* without SDS at *Pe*_e_ = 10 (blue line), with SDS at *Pe*_e_ = 10 (red line), and in the case of SDS-added sphere–ellipsoid binary mixtures (*v*_e_:*v*_s_ = 1) at *Pe*_e_ = 15 (green line).

We computed the alignment *S*^†^ between the ellipsoidal particles along the direction of their short axis (lateral ordering) and along their long axis (longitudinal ordering). All systems exhibited weak longitudinal ordering that decayed very quickly, while the lateral order was much more pronounced and persisted over several particle diameters (Figure S13). Figure 7 shows the lateral ordering of ellipsoids with λ = 7. In the systems without SDS, *S*^†^ decayed rather quickly and reached zero for pairs of ellipsoids that were about seven particle diameters apart (blue line in Figure 7). However, when SDS was added, the lateral ordering of particles on the supraparticle surface improved dramatically, as shown by the red line in Figure 7.

Inside the SDS-added supraparticles (dried at *Pe*_e_ = 10), the ellipsoidal colloids exhibited some local nematic order, which contrasts with the random ellipsoid orientations observed in previous experiments^48^ of supraparticles prepared at 40 ≤ *Pe*_e_ ≤ 70 (with the final packing fraction of the colloids ranging between 0.52 and 0.61). The nematic order arises from entropy-driven organization in liquid-crystalline phases, a concept first introduced by Onsager.^71^ According to Onsager’s theory, hard rods may align in parallel at sufficiently high concentrations when the resulting gain in translational entropy outweighs the loss in orientational entropy. For short rigid rods with λ ≈ 5, the isotropic–nematic transition is weakly first-order and has been shown through Monte Carlo simulations to produce small nematic clusters, each with its own orientation.^72^ For longer rigid rods, the transition is strongly first-order, resulting in a nematic phase with nearly uniform orientation.^72^ We note here that Onsager’s theory describes bulk systems in equilibrium, so it cannot fully capture the nonequilibrium behavior of drying droplets, where the realignment of the ellipsoidal colloids could be hindered at higher evaporation rates and through confinement.

**Figure 8:**
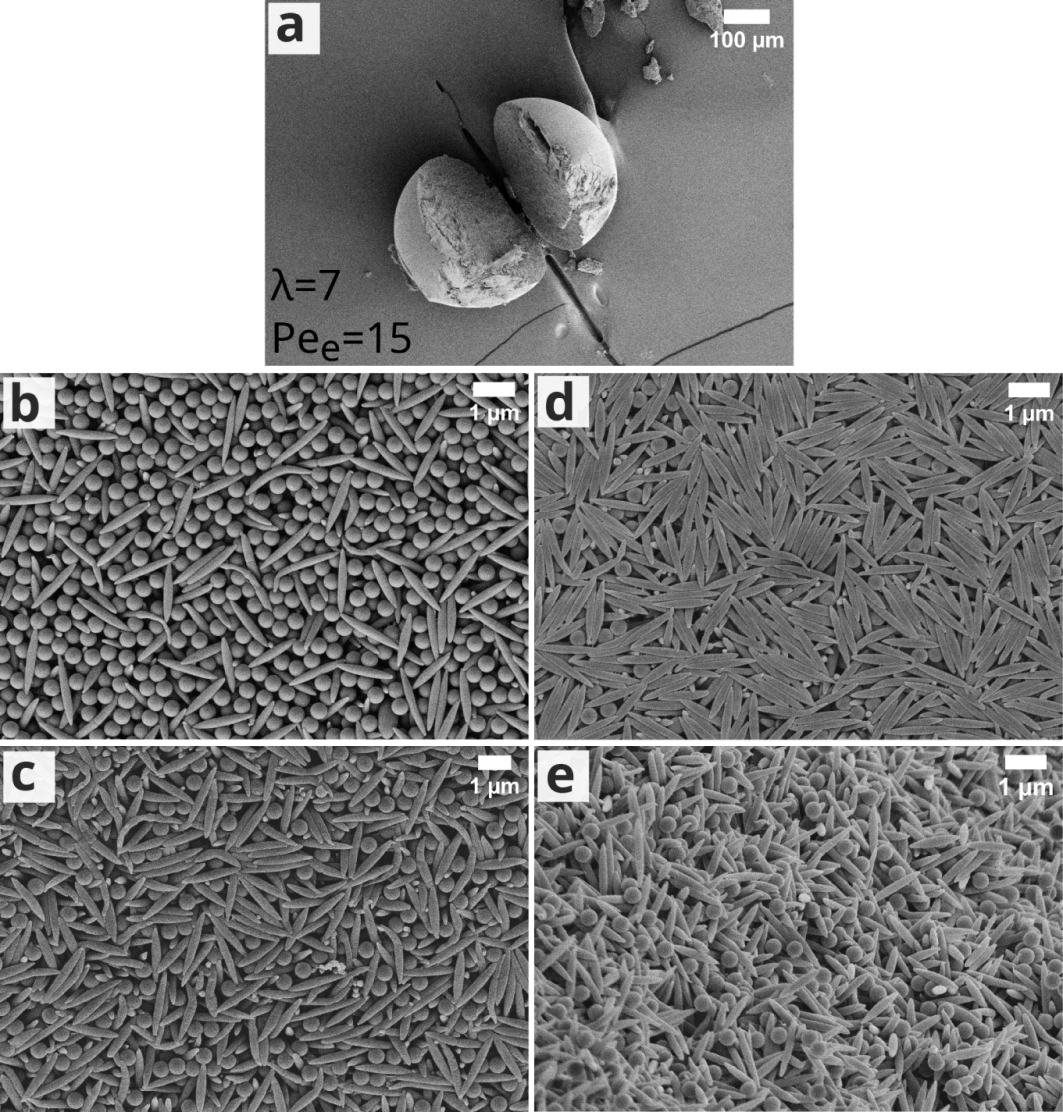
SEM images showing (a) a final supraparticle and (b–d) its surface for sphere–ellipsoid mixtures (λ = 7) when *v*_e_:*v*_s_ = (b) 0.4, (c) 1, (d) 2.5. (e) Supraparticle cross-section for *v*_e_:*v*_s_ = 2.5. The supraparticles were prepared at *Pe*_e_ = 15 and *Pe*_s_ = 5.

Finally, we analyzed whether the addition of spherical colloids affects the translational and orientational order of the ellipsoidal colloids. To this end, we prepared supraparticles containing SDS-added sphere–ellipsoid mixtures (λ = 7 ellipsoids, *Pe*_e_ = 15 and *Pe*_s_ = 5, RH = 90%) at different volume ratios of the components (*v*_e_:*v*_s_ = 0.4, 1, 2.5, 5, and 10). The number of ellipsoidal and spherical colloids on the supraparticles’ surface were counted from the SEM images to estimate the relative area fractions of ellipsoids (σ_e_) and spheres (σ_s_). The supraparticles formed an almost spherical final shape in all cases (Figure 8a). For *v*_e_:*v*_s_ = 0.4, the supraparticle surface was mostly covered by spherical colloids (σ_s_ = 79%) without crystalline order, with ellipsoidal colloids filling the interstitial spaces (Figure 8b). At an equal volume ratio of the components (*v*_e_:*v*_s_ = 1), the supraparticle surface was predominantly occupied by ellipsoidal colloids (σ_e_ = 86%), as shown in Figure 8c. For the systems with *v*_e_:*v*_s_ = 2.5, 5, and 10, the supraparticle surfaces were covered almost exclusively by ellipsoidal colloids, with 92% ≤ σ_e_ ≤ 99% (Figures 8d, S14, and S15). The surface ordering of the ellipsoidal colloids in binary supraparticles was reduced compared to that in pure ellipsoid supraparticles, as shown by the green line in Figure 7 for the case of *v*_e_:*v*_s_ = 1.

Inside the supraparticles, the local nematic ordering of ellipsoidal colloids was fully suppressed by the presence of the spherical colloids, which agrees with our previous drying simulations^48^ that showed only small nematic bundles of rods along with some randomly packed spheres in the diffusion-limited regime (*Pe*_e_ = 1). Ellipsoids and spheres were randomly distributed (Figures 8e, S14, S15, and S16–S18) at all volume ratios of the components and did not phase separate in the interior. Phase separation might be hindered by the nonequilibrium state of the drying droplet and by the spherical confinement that can lead to different behavior compared to the bulk.^73,74^

## Conclusions

We investigated the formation of supraparticles from drying dispersion droplets containing ellipsoids and sphere–ellipsoid binary mixtures using various ellipsoid aspect ratios. We used superamphiphobic surfaces to achieve nearly spherical droplets and controlled the evaporation rate through the relative humidity. All experiments were performed in the evaporation-limited regime (*Pe*_e_ ≫ 1), where the droplet–air interface moves faster than the colloids diffuse, potentially causing steep concentration gradients within the droplet.

In our first set of experiments without surfactants, buckled supraparticles consistently formed regardless of the colloid shape, initial colloid volume fraction, or evaporation rate. We attribute this buckling to the formation of an elastic colloid shell at the droplet–air interface, which ultimately collapsed to relieve the internal stresses. Due to the strong lateral capillary interactions exhibited by ellipsoidal colloids, dependent on their aspect ratio and contact angle, these anisotropic particles may enhance shell deformation during evaporation. This hypothesis aligns with our observation that droplets containing only spherical colloids produced less deformed supraparticles.

We were able to suppress buckling by adding surfactant to the droplets, which likely increased the electrostatic double-layer repulsion between colloids and thus delayed shell formation. At evaporation rates approaching the diffusion-limited regime, the ellipsoids exhibited increased ordering both on the supraparticle surface and within its interior, consistent with our previous simulation results.^48^ Overall, the degree of ordering increased with slower drying speeds.

For sphere–ellipsoid mixtures, the local nematic ordering of ellipsoidal colloids within the supraparticles was suppressed, leading to a random distribution of spherical and ellipsoidal colloids. Phase separation during the drying process of binary mixtures could be influenced by factors, such as the degree of confinement, initial colloid volume fractions, and colloid sizes. This phase separation behavior can be harnessed to produce multicomponent supraparticles with diverse architectures such as core–shell or Janus structures.

## Supporting Information Available

Confocal videos showing the morphology of an evaporating dispersion droplet containing sphere–ellipsoid mixtures (ZIP)
Video tracking the morphology of an evaporating dispersion droplet containing ellipsoids that leads to buckling (MP4)
Additional SEM images of supraparticles and schematics showing the buckling process during evaporation (PDF)
