# Supplementary material for: Tuning the Morphology and Structure of Supraparticles Composed of Ellipsoids
Source: Langmuir. 2025 Mar 10;41(11):7845–55. doi: 10.1021/acs.langmuir.5c00409 (PMC11948468; doi:10.1021/acs.langmuir.5c00409)
Supplement: Supplementary file 3 — la5c00409_si_003.pdf [file la5c00409_si_003.pdf]

Supporting Information: Tuning the Morphology and Structure of Supraparticles Composed of Ellipsoids

Melis Yetkin,[†] Yashraj Manish Wani,[‡] Arash Nikoubashman,[¶,§] Hans-Jürgen Butt,[†] and Michael Kappl[*,†]

†*Department of Physics at Interfaces, Max-Planck Institute for Polymer Research, Mainz 55128, Germany*

‡*Institute of Physics, Johannes Gutenberg University of Mainz, Staudingerweg 7, 55128 Mainz, Germany*

¶*Leibniz-Institut für Polymerforschung Dresden e.V., Hohe Straße 6, 01069 Dresden, Germany*

§*Institut für Theoretische Physik, Technische Universität Dresden, 01069 Dresden, Germany*

E-mail: kappl@mpip-mainz.mpg.de

Péclet number calculation

Evaporation of dispersion droplets is a non-equilibrium transport process in confinement, which can be characterized by the dimensionless Péclet number, Pe. Here, we define Pe as the ratio of the typical diffusion time of the colloidal particles, τ_d, and the characteristic evaporation time of the droplets, τ_{ev}.

For a water droplet evaporating at a constant contact angle, the droplet's surface $V^{2/3}$

decreases linearly with time as[1]

$$V^{2/3} = V_0^{2/3} - \alpha t \tag{1}$$

where V_0 is the initial volume of the droplet and α characterizes the (constant) rate of change of the droplet's surface area. Colloidal droplets evaporate mostly in the same manner, but with a slightly slower rate.[2] The droplet volume was estimated by applying the solid of revolution method on the images obtained from the evaporation videos.[3] Here, we first determined an arc through the circumference of the 2D image of one half of the droplet, and then integrated disc-shaped slices, which passed through this arc and cut the central axis of the droplet perpendicularly around the central axis. The method assumed no predefined droplet shape.

By knowing the droplet volume at each time, we can extract α by fitting our experimental data to equation (1). The initial speed of the droplet interface, v_{ev}, is related to α through[2]

$$v_{ev} = \frac{1}{2}\left(\frac{3}{4\pi}\right)^{2/3}\frac{\alpha}{R_0} \tag{2}$$

where R_0 is the initial droplet radius. Then, we calculated the characteristic evaporation time as $\tau_{ev} = R_0/v_{ev}$.

The diffusion time is defined as $\tau_d = R_0^2/D_0$, with D_0 being the translational diffusion coefficient of the colloidal particles at infinite dilution. For a spherical colloid, D_0 can be estimated through the Stokes-Einstein relation as follows

$$D_0 = \frac{k_B T}{3\pi\eta d} \tag{3}$$

where η is the medium's viscosity and d is the diameter of the spherical colloid. For the sake of simplicity, the viscosity was taken as that of water ($\eta = 10^{-3}\,\mathrm{Pa\,s}$).

For an ellipsoid, the parallel diffusion coefficient ($D_{\parallel}$) and the perpendicular diffusion

coefficient ($D_\perp$) can be estimated as[4]

$$D_\parallel = \frac{k_B T ln(L/d_e)}{2\pi\eta L} \tag{4}$$

$$D_\perp = \frac{k_B T ln(L/d_e)}{4\pi\eta L} \tag{5}$$

Using these expressions for $D_\parallel$ and $D_\perp$, the diffusion coefficient for an ellipsoidal colloid D_e is found by[4]

$$D_e = \frac{(D_\perp + 2D_\parallel)}{3} \tag{6}$$

The final expression for Pe then reduces down to

$$Pe = \frac{\tau_d}{\tau_{ev}} = \frac{v_{ev} R_0}{D_0} \tag{7}$$

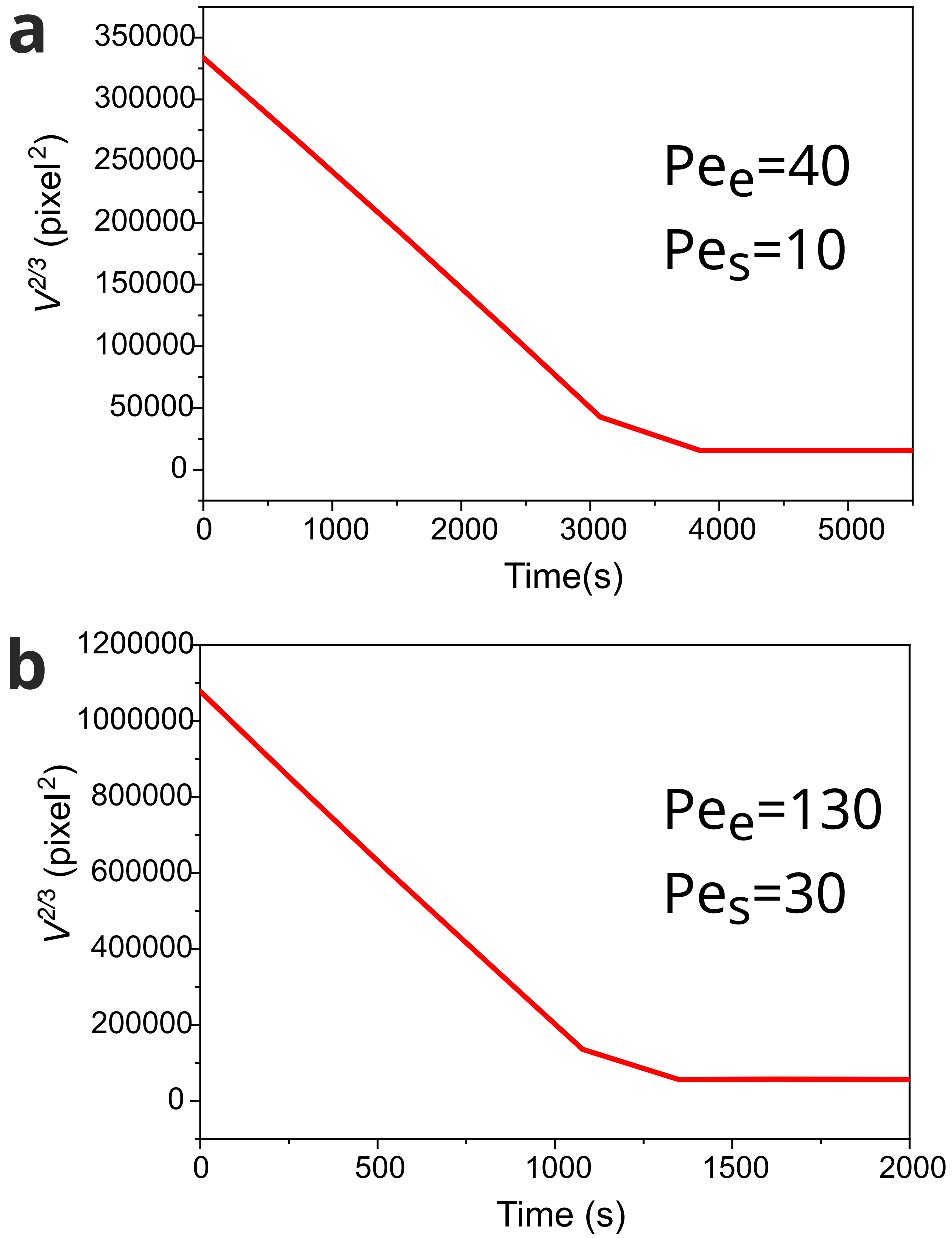

Figure S1: Evaporation curves showing the almost linear decrease of droplet $V^{2/3}$ over time for sphere-ellipsoid ($\lambda = 4$) mixtures at RH= (a) 75% and b) (30%. The Péclet numbers for ellipsoids (Pe_e) and spheres (Pe_s) are labelled on the plots.

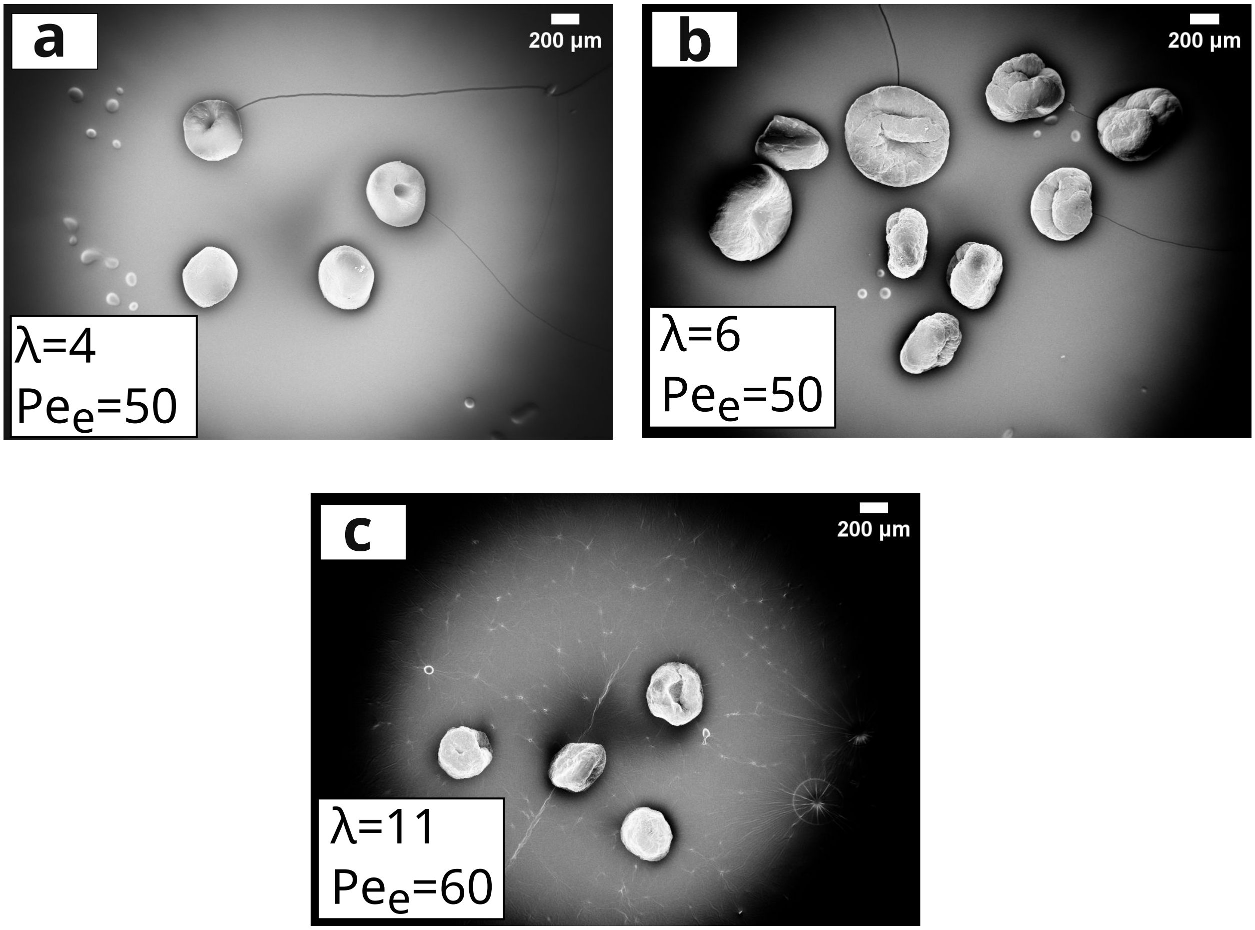

Figure S2: SEM images of the final buckled morphology of supraparticles composed of ellipsoids of (a) $\lambda = 4$, (b) $\lambda = 6$, and (c) $\lambda = 11$. The evaporation experiments were performed at RH=75%.

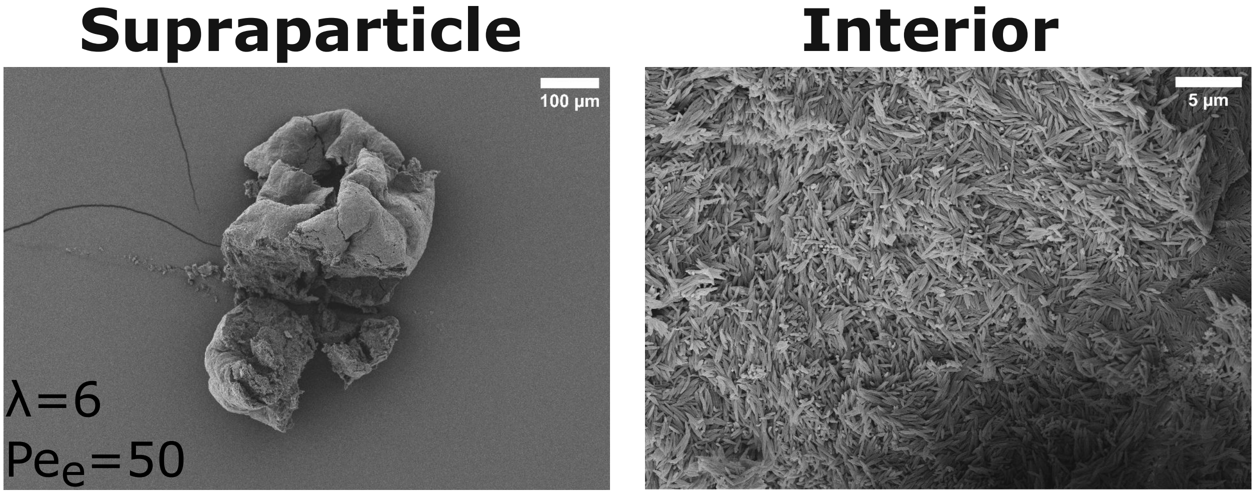

Figure S3: SEM images of a broken supraparticle (left) and its cross-section (right) composed of $\lambda = 6$ ellipsoids dried at $Pe_e = 50$.

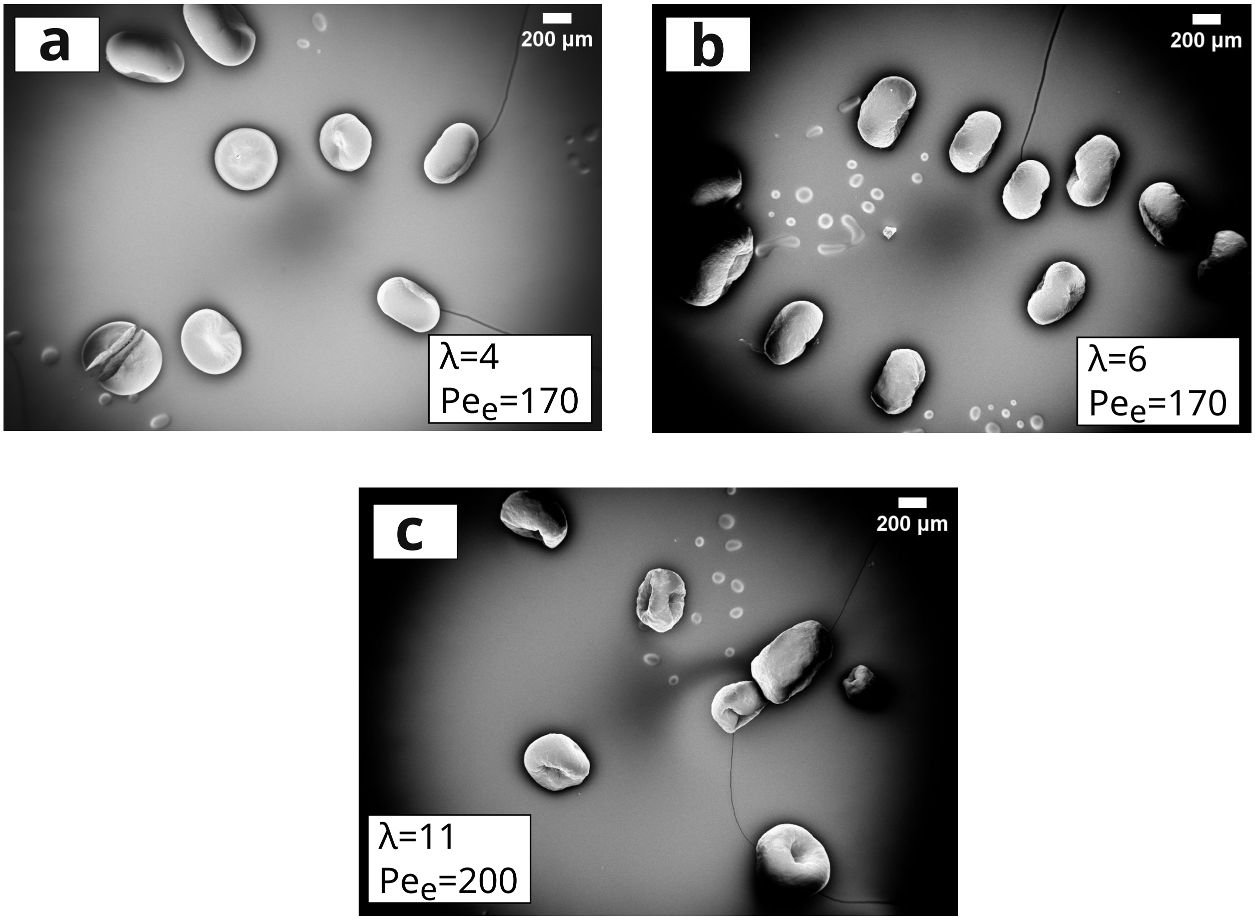

Figure S4: SEM images of the final buckled morphology of supraparticles composed of ellipsoids of (a) $\lambda = 4$, (b) $\lambda = 6$, and (c) $\lambda = 11$. The evaporation experiments were performed at RH=30%.

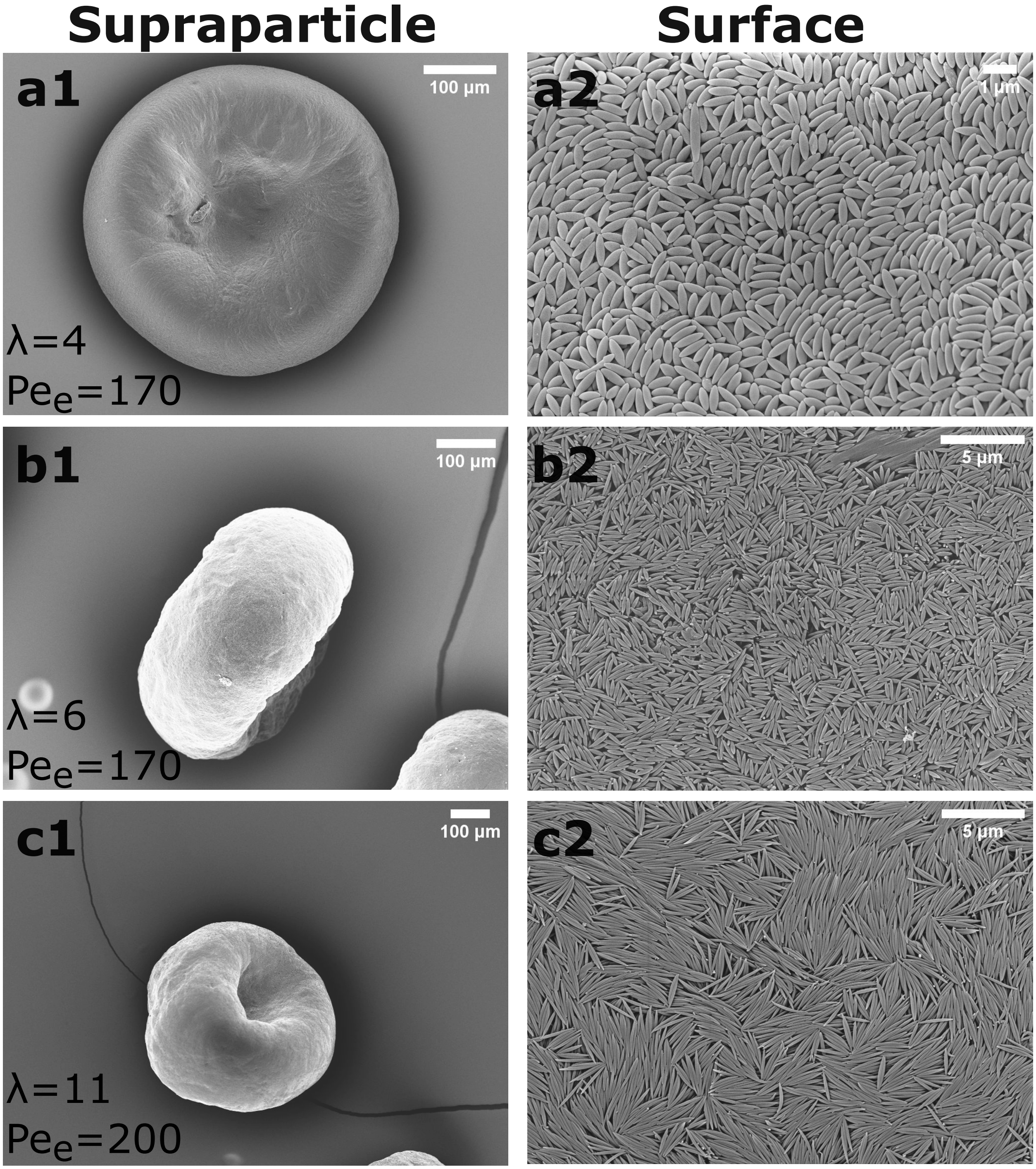

Figure S5: SEM images showing the supraparticles and their corresponding surfaces, comprising ellipsoids of (a1,a2) $\lambda = 4$, (b1,b2) $\lambda = 6$, and (c1,c2) $\lambda = 11$ dried at $170 \leq Pe_e \leq 200$.

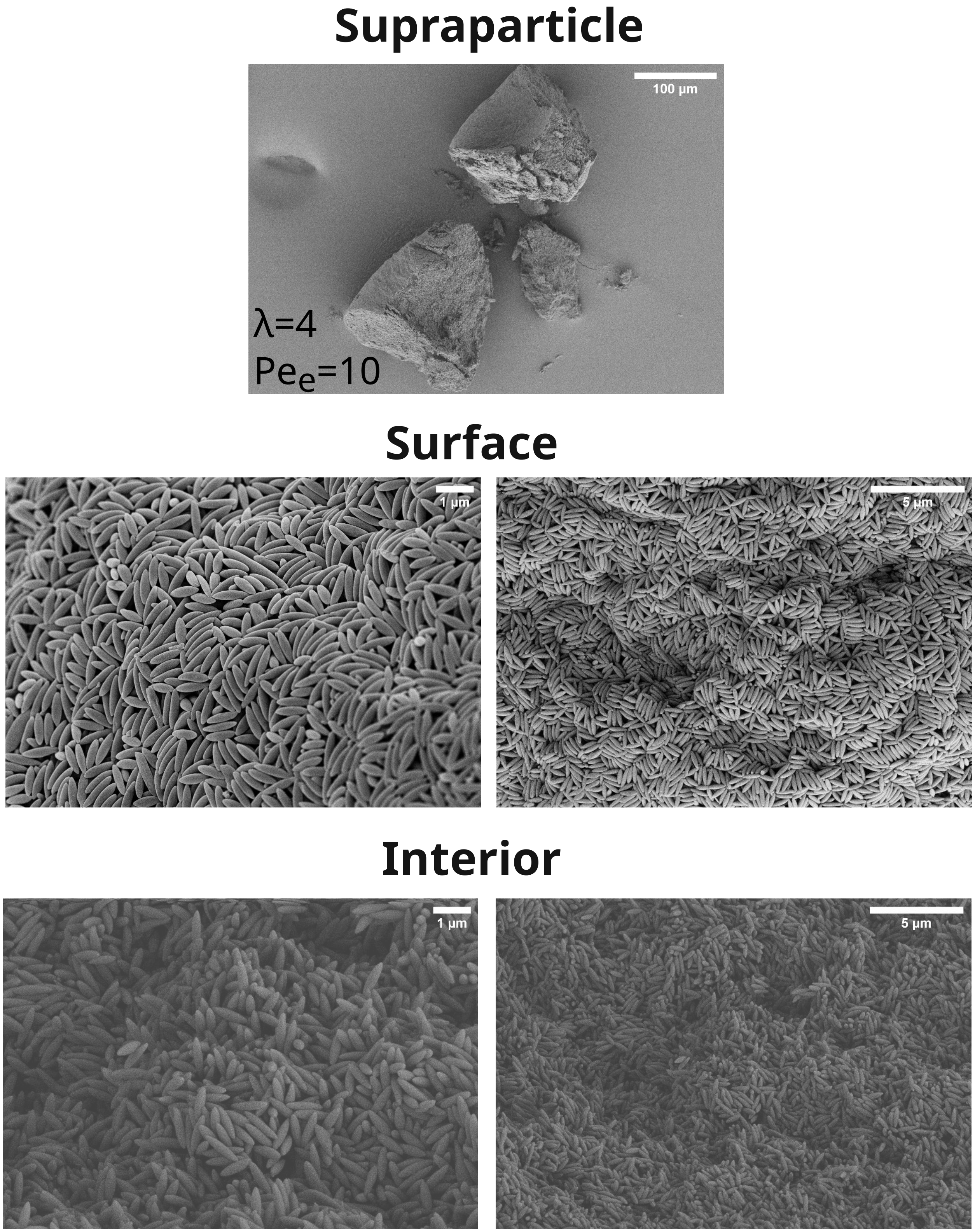

Figure S6: SEM images showing a broken supraparticle (top), its surface (middle), and its cross-section (bottom), composed of $\lambda = 4$ ellipsoids dried at $Pe_e = 10$.

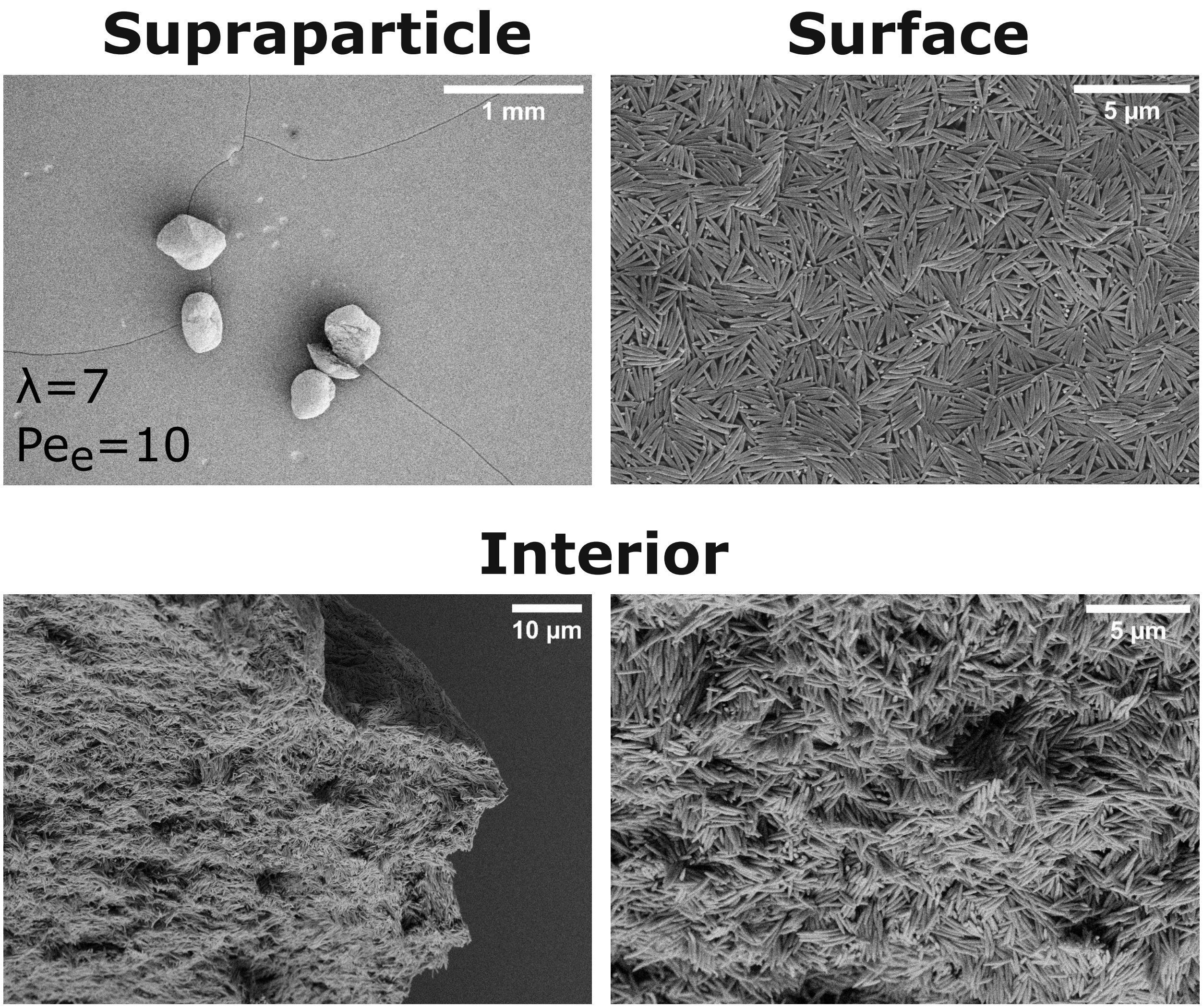

Figure S7: SEM images showing supraparticles, the supraparticle surface, and supraparticle cross-sections composed of $\lambda = 7$ ellipsoids dried at $Pe_e = 10$.

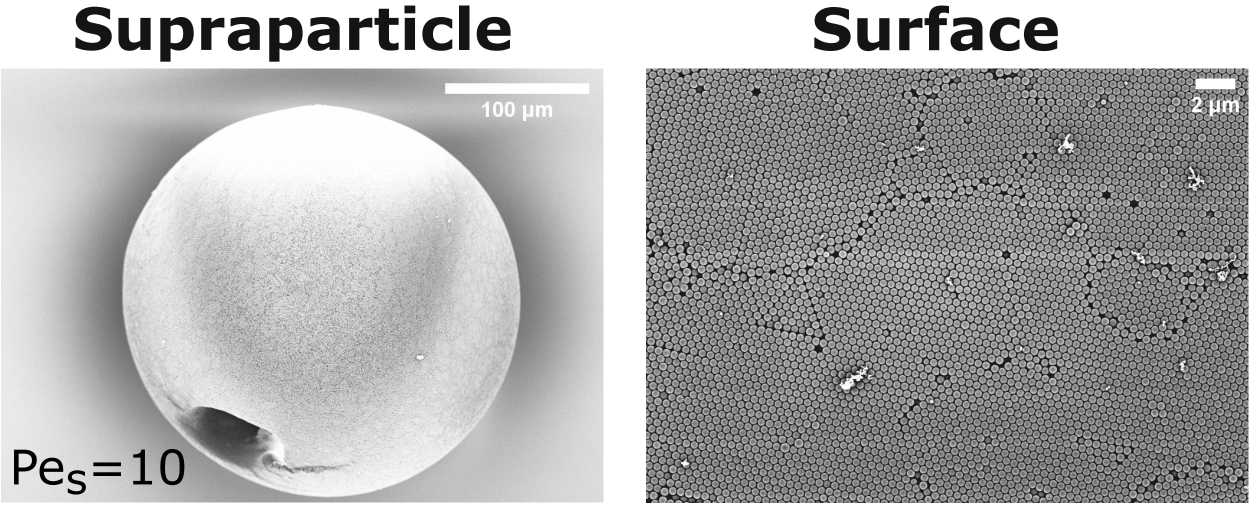

Figure S8: SEM images of a supraparticle (left) and its surface (right) composed of PS spheres dried at $Pe_s = 10$.

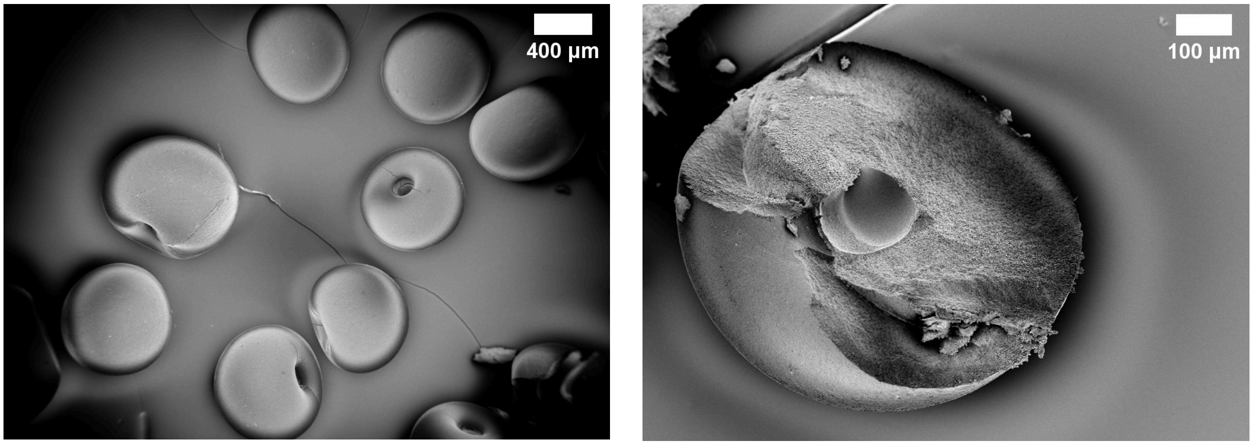

Figure S9: SEM images of supraparticles composed of PS spheres with increased initial volume fraction (8% initial volume fraction). The SEM image (right) shows a broken supraparticle.

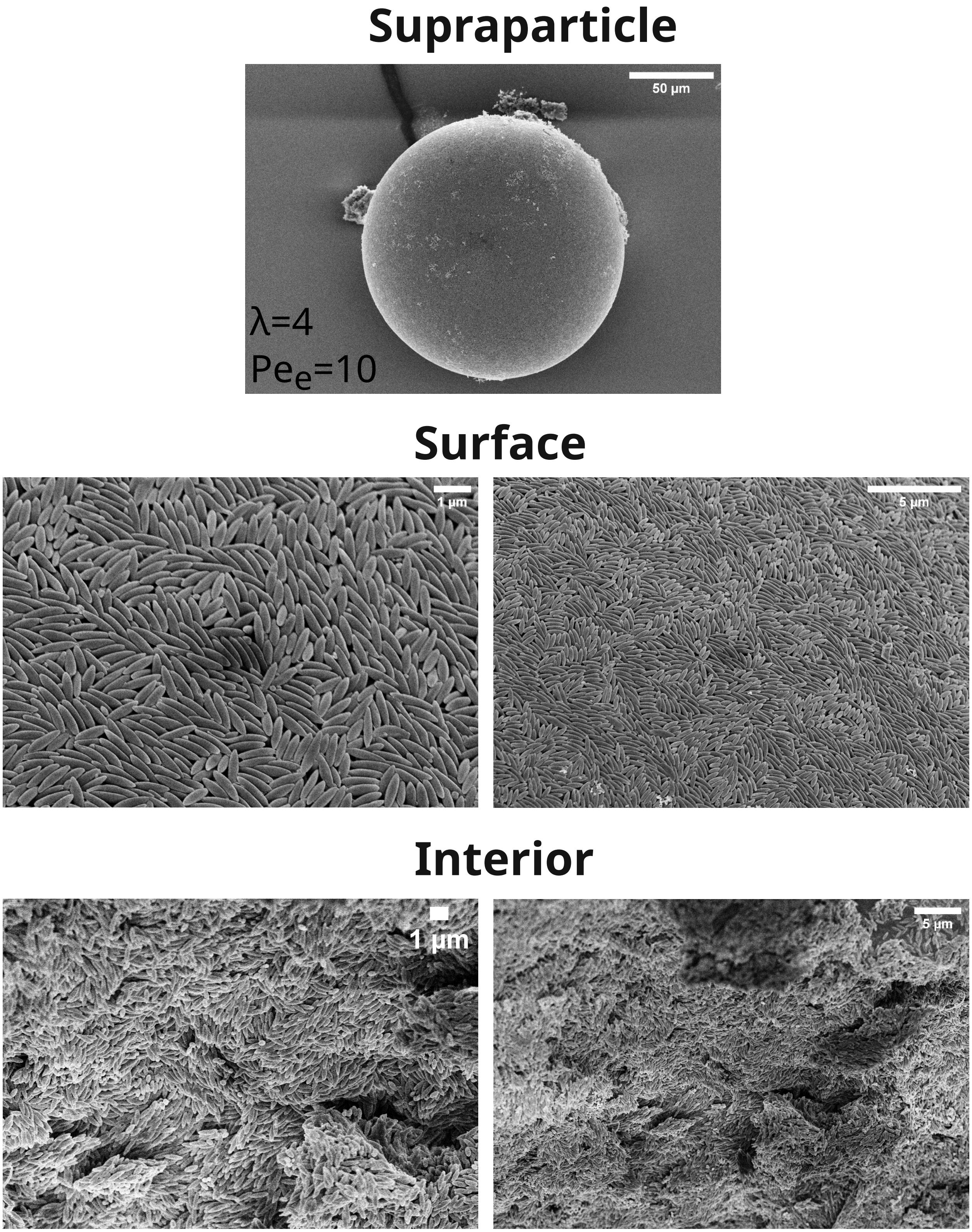

Figure S10: SEM images showing a supraparticle (top), its surface (middle), and its cross-section (bottom) composed of $\lambda = 4$ ellipsoids with SDS dried at $Pe_e = 10$.

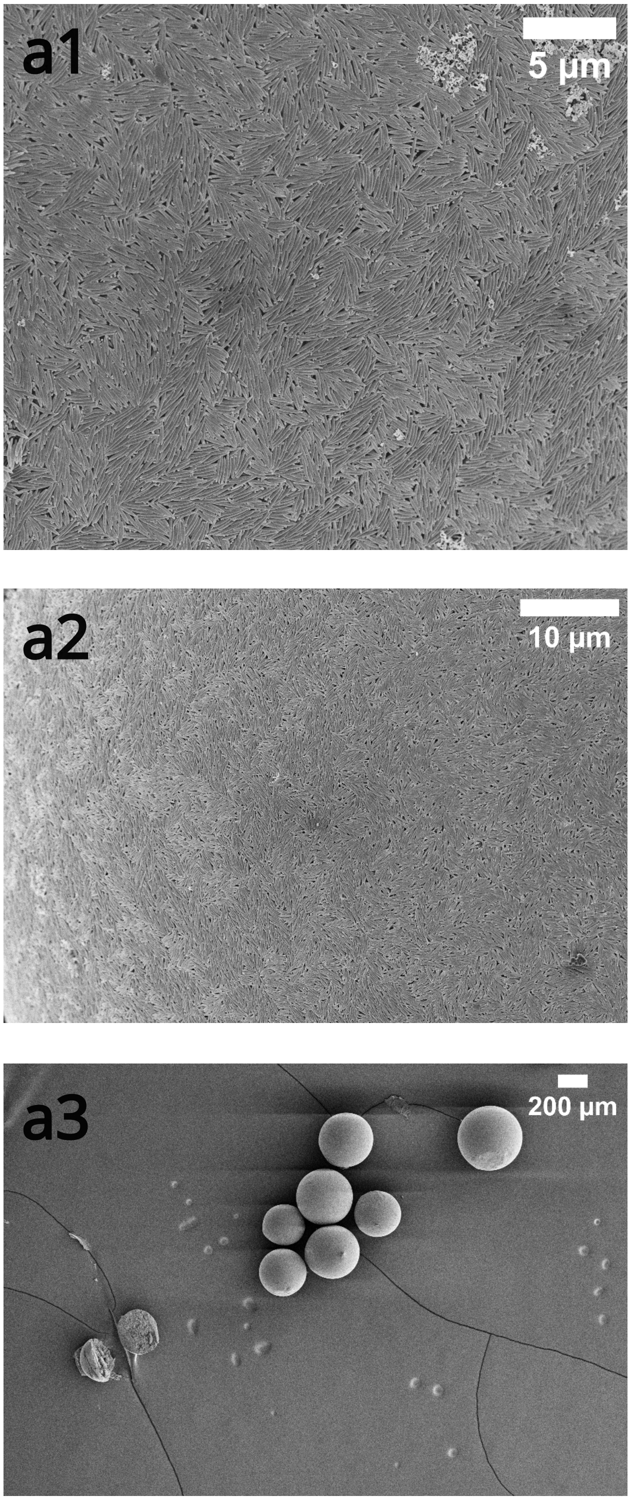

Figure S11: SEM images showing (a1,a2) the supraparticle surface at two different magnifications, and (a3) many supraparticles composed of $\lambda = 7$ ellipsoids with SDS dried at $Pe_e = 10$.

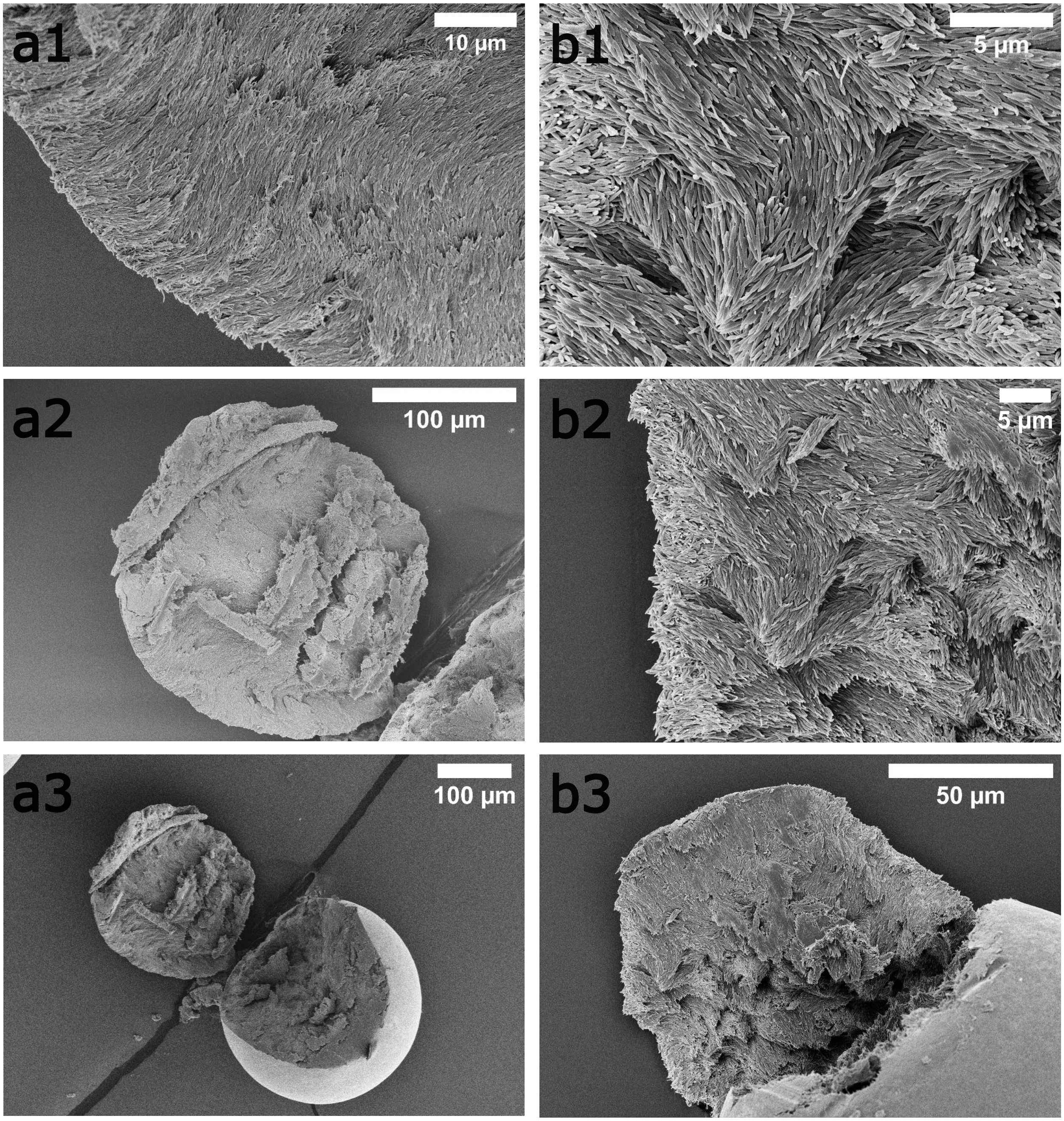

Figure S12: SEM images showing the cross-sections of two supraparticles composed of $\lambda = 7$ ellipsoids with SDS dried at $Pe_e = 10$. Images were obtained by zooming out from the same region of interest from (a1-a3) and from (b1-b3).

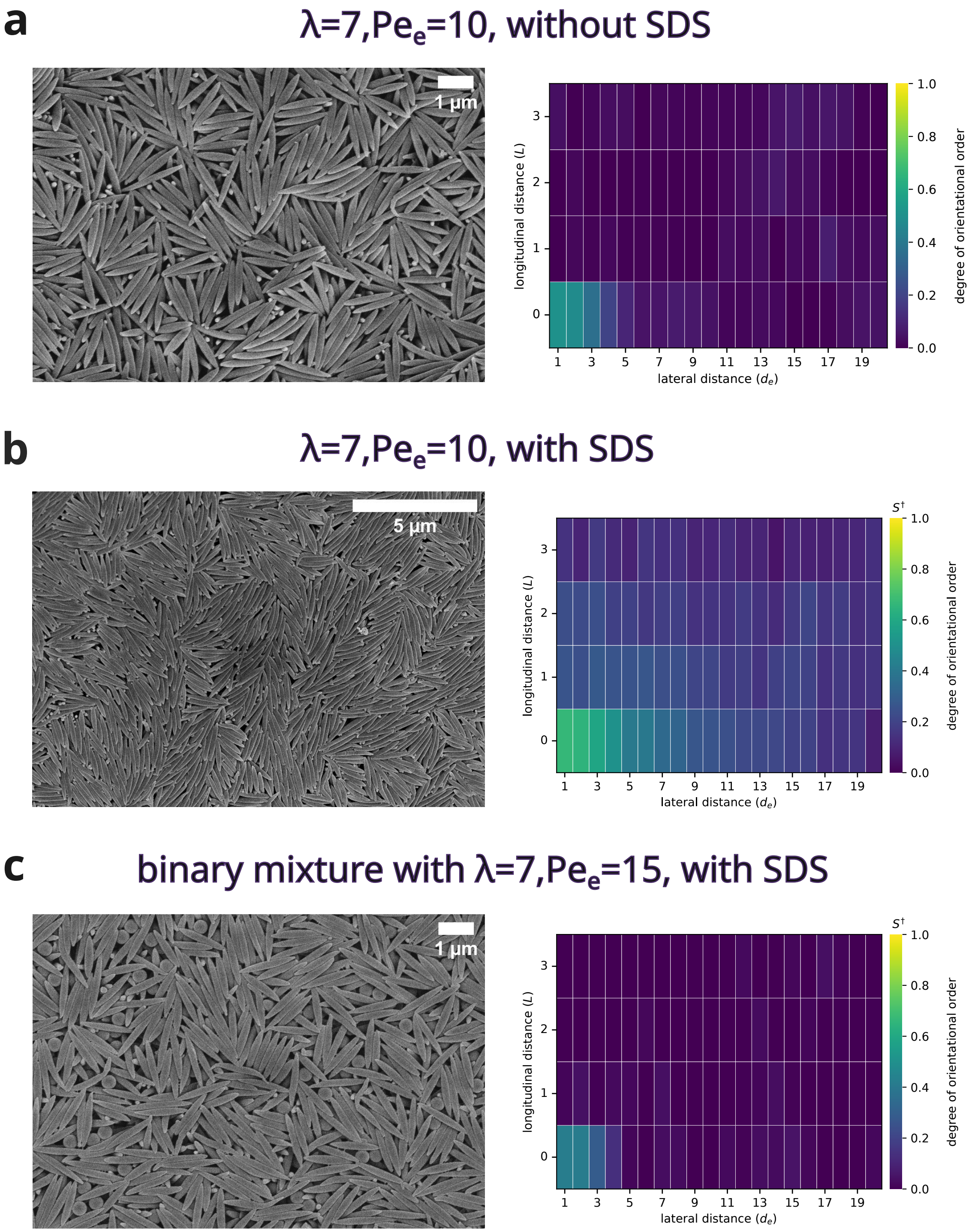

Figure S13: 2D heat map representation of the orientational order parameter $S^{\dagger}$ with respect to lateral and longitudinal distance around ellipsoids with $\lambda = 7$. The data were computed from the SEM images shown left for supraparticles (a) without SDS dried at $Pe_e = 10$, (b) with SDS dried at $Pe_e = 10$, and (c) containing sphere-ellipsoid mixtures (v_e:v_s = 1) with SDS dried at $Pe_e = 15$.

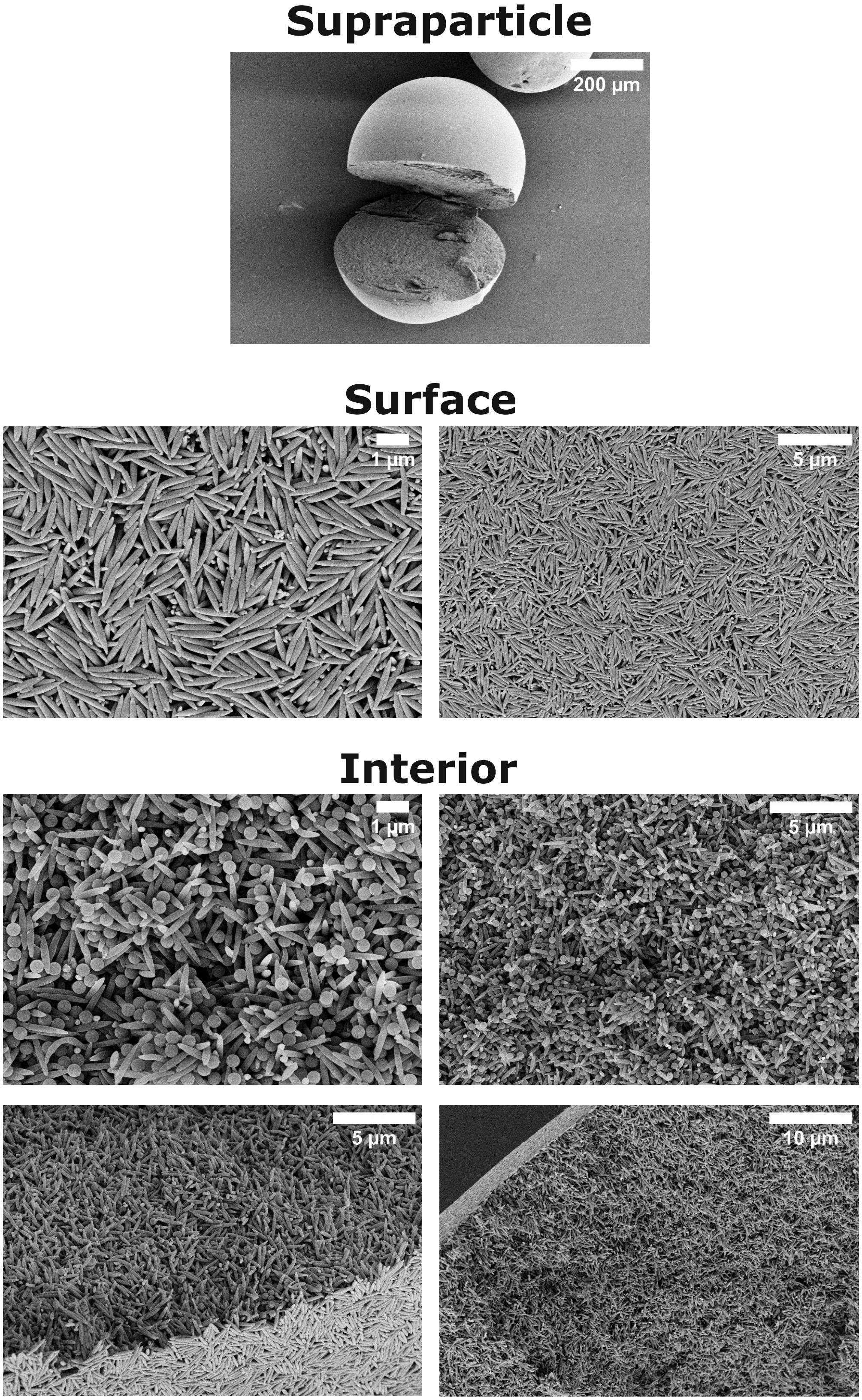

Figure S14: SEM images showing a supraparticle (top), its surface (middle), and its cross-section (bottom) composed of sphere-ellipsoid mixtures with $\lambda = 7$ and v_e:$\mathrm{v}_s = 5$. The supraparticles were obtained at $Pe_e = 15$ and $Pe_s = 5$.

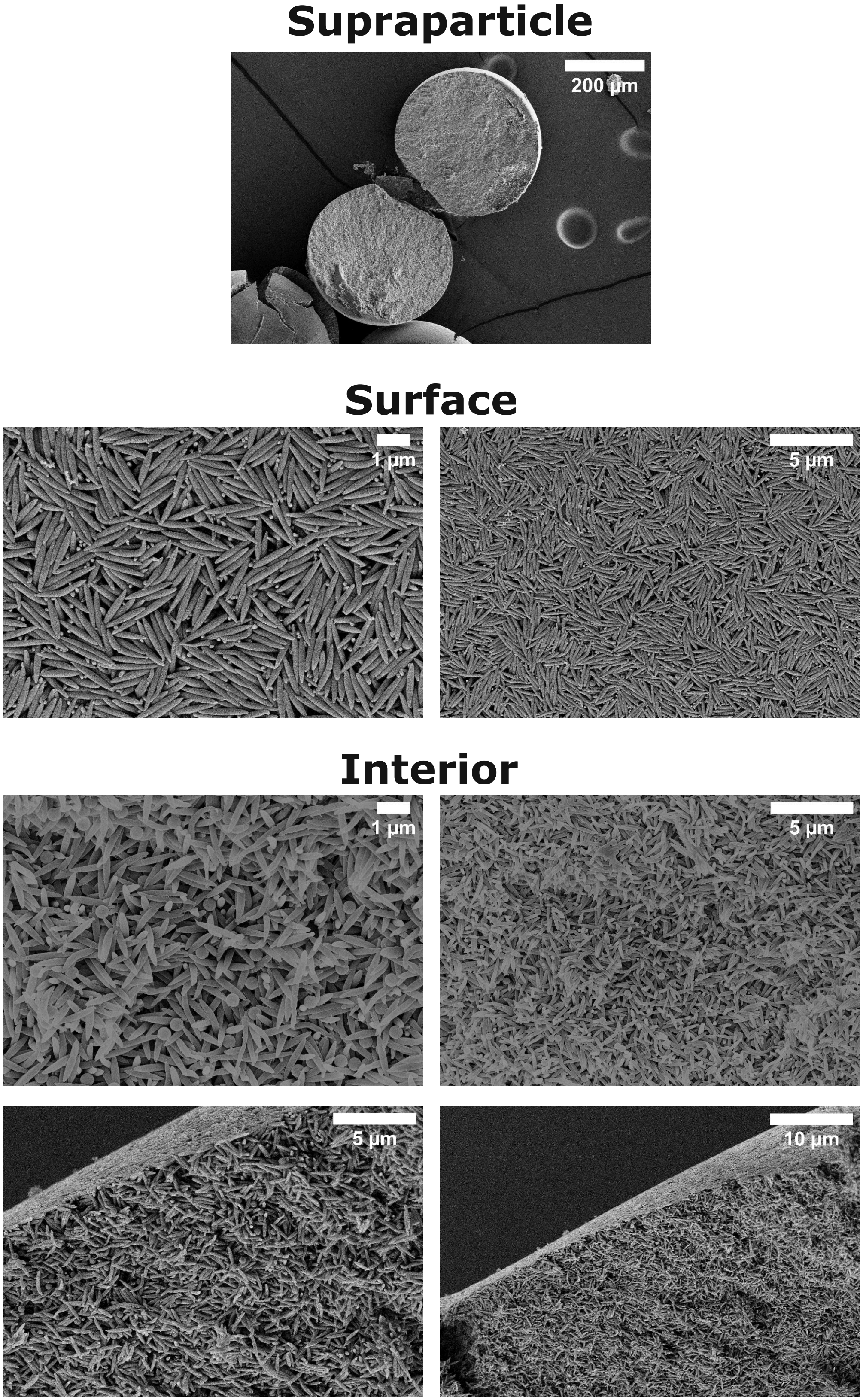

Figure S15: SEM images showing a supraparticle (top), its surface (middle), and its cross-section (bottom) composed of sphere-ellipsoid mixtures with $\lambda = 7$ and v_e:$\mathrm{v}_s = 10$. The supraparticles were obtained at $Pe_e = 15$ and $Pe_s = 5$.

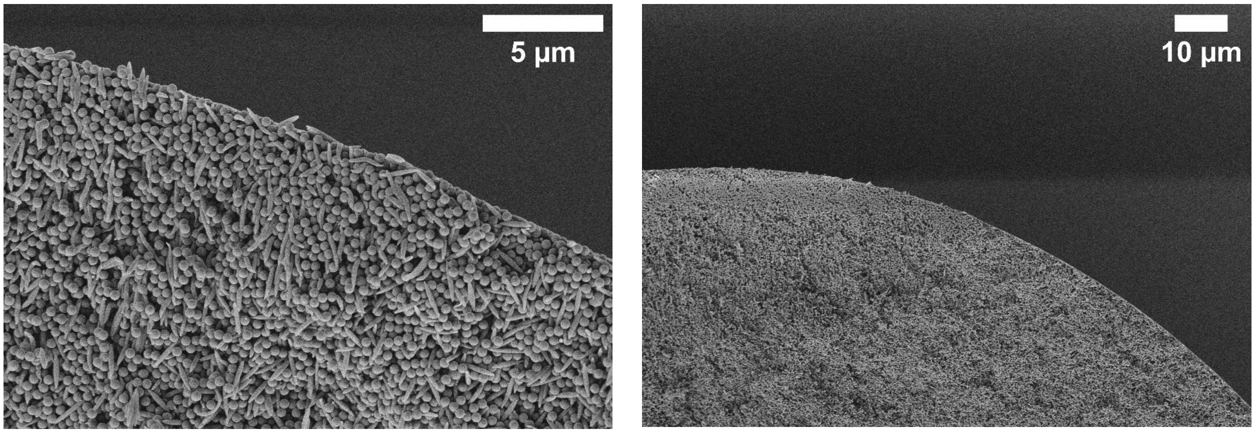

Figure S16: SEM images showing the cross-section of supraparticles composed of sphere-ellipsoid mixtures with $\lambda = 7$ and v_e:$\mathrm{v}_s = 0.4$. The supraparticles were obtained at $Pe_e = 15$ and $Pe_s = 5$. Images were obtained by zooming out from the same region of interest from left to right.

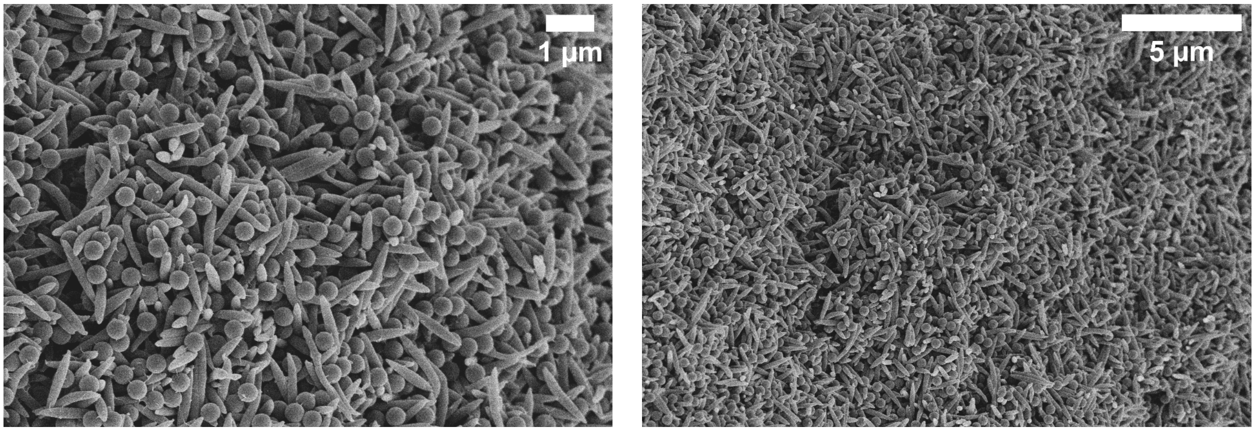

Figure S17: SEM images showing the cross-section of supraparticles composed of sphere-ellipsoid mixtures with $\lambda = 7$ and v_e:$\mathrm{v}_s = 1.0$. The supraparticles were obtained at $Pe_e = 15$ and $Pe_s = 5$. Images were obtained by zooming out from the same region of interest from left to right.

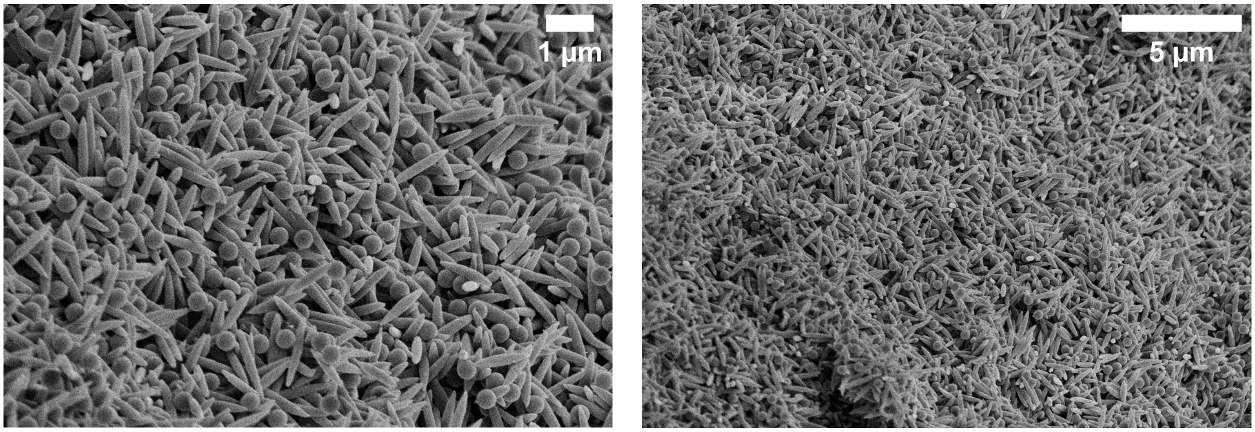

Figure S18: SEM images showing the cross-section of supraparticles composed of sphere-ellipsoid mixtures with $\lambda = 7$ and v_e:$\mathrm{v}_s = 2.5$. The supraparticles were obtained at $Pe_e = 15$ and $Pe_s = 5$. Images were obtained by zooming out from the same region of interest from left to right.

References

[1]Picknett, R.G. and Bexon, R. The evaporation of sessile or pendant drops in still air. Journal of Colloid and Interface Science 1977, 61(2):336–350.

[2]Liu, W.; Midya, J.; Kappl, M.; Butt, H.-J.; Nikoubashman, A. Segregation in Drying Binary Colloidal Droplets. ACS Nano 2019, 13, 4972–4979.

[3]Yetkin, M.; Wani, Y. M.; Kritika, K.; Howard, M. P.; Kappl, M.; Butt, H.-J.; Nikoubashman, A. Structure Formation in Supraparticles Composed of Spherical and Elongated Particles. Langmuir 2024, 40, 1096–1108.

[4]Vasanthi, R.; Bhattacharyya, S.; Bagchi, B. Anisotropic diffusion of spheroids in liquids: Slow orientational relaxation of the oblates. The Journal of Chemical Physics 2002, 116(3):1092–1096.
